# Melatonin Reduces Neuroinflammation and Improves Axonal Hypomyelination by Modulating M1/M2 Microglia Polarization via JAK2-STAT3-Telomerase Pathway in Postnatal Rats Exposed to Lipopolysaccharide

**DOI:** 10.1007/s12035-021-02568-7

**Published:** 2021-09-28

**Authors:** Qiuping Zhou, Lanfen Lin, Haiyan Li, Huifang Wang, Shuqi Jiang, Peixian Huang, Qiongyu Lin, Xuan Chen, Yiyu Deng

**Affiliations:** 1 Department of Critical Care Medicine, Guangdong Provincial People’s Hospital, Guangdong Academy of Medical Sciences, Guangzhou, 510080 China; 2 School of Medicine, South China University of Technology, Guangzhou, 510006 China; 3 Department of Critical Care Medicine, Guangdong Second Provincial General Hospital, Guangzhou, 510317 Guangdong China; 4 The Second School of Clinical Medicine, Southern Medical University, Guangzhou, 510515 China; 5 Department of Critical Care Medicine, Jieyang People’s Hospital, Jieyang, 522000 Guangdong China; 6 Shantou University Medical College (FCS), Shantou, 515063 China

**Keywords:** Periventricular white matter damage, Melatonin, Microglial polarization, Axonal hypomyelination, JAK2, STAT3, Telomerase reverse transcriptase

## Abstract

Microglia activation and associated inflammation are implicated in the periventricular white matter damage (PWMD) in septic postnatal rats. This study investigated whether melatonin would mitigate inflammation and alleviate the axonal hypomyelination in the corpus callosum in septic postnatal rats. We further explored if this might be related to the modulation of microglial polarization from M1 phenotype to M2 through the JAK2/STAT3/telomerase pathway. We reported here that indeed melatonin not only can it reduce the neurobehavioral disturbances in LPS-injected rats, but it can also dampen microglia-mediated inflammation. Thus, in LPS + melatonin group, the expression of proinflammatory mediators in M1 phenotype microglia was downregulated. As opposed to this, M2 microglia were increased which was accompanied by upregulated expression of anti-inflammatory mediators along with telomerase reverse transcriptase or melatonin receptor 1(MT1). In parallel to this was decreased NG2 expression but increased expression of myelin and neurofilament proteins. Melatonin can improve hypomyelination which was confirmed by electron microscopy. In vitro in primary microglia stimulated by LPS, melatonin decreased the expression of proinflammatory mediators significantly; but it increased the expression of anti-inflammatory mediators. Additionally, the expression levels of p-JAK2 and p-STAT3 were significantly elevated in microglia after melatonin treatment. Remarkably, the effect of melatonin on LPS-treated microglia was blocked by melatonin receptor, JAK2, STAT3 and telomerase reverse transcriptase inhibitors, respectively. Taken together, it is concluded that melatonin can attenuate PWMD through shifting M1 microglia towards M2 via MT1/JAK2/STAT3/telomerase pathway. The results suggest a new therapeutic strategy whereby melatonin may be adopted to convert microglial polarization from M1 to M2 phenotype that would ultimately contribute to the attenuation of PWMD.

**Supplementary Information:**

The online version contains supplementary material available at 10.1007/s12035-021-02568-7.

## Introduction

Sepsis is recognized as one of the main causes of infant death. Although significant progress has been made in recent years in postnatal care and novel antibiotic agents, sepsis remains the main cause of mortality and morbidity in neonates [1]. Sepsis is characterized as uncontrolled inflammatory responses due to proven bacterial infection [2]. Treatment of postnatal sepsis can be achieved as empirical therapy, directed therapy and adjunctive therapy [1]. In view of the recent advances in perinatal management of high-risk premature infants, the recovery rate of postnatal sepsis has improved significantly. However, the improvement of neurological impairment is still unsatisfactory. This may be attributed to the lack of a fuller understanding of the underlying molecular mechanism of neuroinflammation that is implicated in different neuropathological changes in postnatal sepsis such as the periventricular white matter damage (PWMD) [3, 4], cerebral palsy [5, 6] and cognitive and affective disorders [7, 8]. A hallmark neuropathological lesion in postnatal sepsis is diffuse PWMD. There is mounting evidence indicating an intense correlation between PWMD and long-term neurological disorders [9–12], yet attempts to develop related therapies that can promote functional recovery following PWMD remain elusive. The vulnerability of oligodendrocytes to neuroinflammation as elicited by microglial activation and the resulting hypomyelination have been accounted for the main cause of pathogenesis of PWMD [13, 14]. In light of the above, it is reasoned that hypomyelination may be one of the key degenerative mechanisms for PWMD. The differentiation and maturation of oligodendrocyte progenitor cells (OPCs) is described to play a crucial role in axonal remyelination [15–17]. Therefore, promoting the differentiation and maturation of OPCs and reducing microglia induced neuroinflammation may be a prospective therapeutic strategy for mitigation of PWMD and axonal hypomyelination in postnatal sepsis.

Melatonin is a neurohormone that is mainly produced by the pineal gland [18]. A distinctive characteristic of melatonin is its circadian synthesis profile with the environmental light/dark cycle [19]. It is well documented that melatonin is endowed with diverse pharmacological properties such as anti-oxidation, anti-inflammation and anti-apoptotic effects [20–22]. Melatonin can gain access readily into different areas of the central nervous system (CNS) and exerts its functions because it can readily cross the blood–brain-barrier (BBB) [23, 24]. There is ample evidence supporting that melatonin possesses a neuroprotective effect against different neurodegenerative diseases including Alzheimer’s disease, Parkinson’s disease, hypoxic-ischemic induced brain injury. [25–27]. Melatonin receptors are localized in different cell types in the CNS, including microglia, neurons and astrocytes [28, 29]. Studies have indicated that melatonin receptor is highly expressed in microglia and astrocytes; however, it is only moderately expressed in oligodendrocytes [57]. It is therefore reasonable to suggest that melatonin is more likely to act directly on microglia and astrocytes, and less so on oligodendrocytes. We reported previously that activated microglia were instrumental to neuroinflammation and that they were closely associated with PWMD induced by postnatal sepsis [3, 30]; Recent studies have reported that neuroinflammation may induce polarization of reactive microglia, namely, M1 (a classically activated microglial phenotype) and M2 (an alternatively activated microglial phenotype). The M1 phenotype tends to release proinflammatory cytokines such as IL-1β and TNF-α that can aggravate tissue injury; on the other hand, the M2 phenotype preferentially releases anti-inflammatory cytokines and neurotrophic factors such as CD206 and TGF-β that promote inflammation resolution and tissue repair. This invites speculation that modulating microglial polarization towards the M2 phenotype may be a potential therapeutic strategy to reduce neuroinflammation and consequently improve PWMD in postnatal sepsis.

Telomerase is a reverse transcriptase best known for its telomere maintenance function in stem cells [31, 32]; it has two subunits including the telomerase reverse transcriptase (TERT) and the telomerase RNA component (TERC) [33, 34]. Although telomerase activity is mainly localized in areas containing stem cells, studies have shown non-telomeric function of the TERT protein. A high level of telomerase expression has been found in the process of embryonic brain development, and TERT expression is maintained into the adulthood in rodents [34–36]. TERT protein has been detected in cultured neurons and microglia in vitro [37–39]. Very interestingly, transgenic overexpression of TERT exhibited a remarkable resistance to damage. Induction of TERT expression in damaged neurons protected them against NMDA excitotoxicity, and, furthermore, it ameliorated ischemic neuronal death [40]. As opposed to the above, TERT deficiency potentiated ischemia-induced neurological disorders, tissue damage, and BBB dysfunction in comparison with control mice with normal TERT expression [36, 41, 42]. Evidence gained from these studies strongly indicated that TERT expression is important in the CNS especially of its role in neuroprotection. Very interestingly, it has been reported that melatonin can affect the expression of TERT in breast cancer and leukemia [43, 44]. Furthermore, it has been reported that the JAK2/STAT3 pathway participates in the protective effect of erythropoietin (EPO) in subarachnoid hemorrhage (SAH); also, JAK2 and telomerase have a combination effect in the treatment of myeloproliferative neoplasms (MPNs) [45]. Arising from the above studies, our attention was first drawn to the potential relationship between melatonin, telomerase and microglial polarization and their effect on inflammation in postnatal sepsis. This study was therefore undertaken to ascertain if melatonin would ameliorate neuroinflammation in PWMD and, if so, to determine if the effect is through JAK2/STAT3-telomerase pathway by modulating the inflammatory response and microglia polarization. We report here that indeed melatonin is beneficial in mitigation of PWMD through enhancing telomerase expression in activated microglia in postnatal sepsis in rats.

## Materials and Methods

### Animals

One-day-old Sprague–Dawley (SD) rats (*n* = 240) obtained from the Experimental Animal Center of Sun Yat-sen University were used in this study. The rats were housed on standard conditions at 25 ± 1 °C and humidity at 55 ± 5% with a 12-h light/dark alternation. Food and water were supplied to the rats ad libitum. The rats used in this study were randomly divided into three groups: (I) the control group. The rats in this group were intraperitoneally administered with 0.01 M phosphate buffered saline (PBS) (1 mg/kg); (II) LPS group. In this group, rats were intraperitoneally injected with lipopolysaccharide (LPS) (1 mg/kg) taken from *Escherichia coli* O111:B4 (Sigma-Aldrich, L4391); (III) LPS + melatonin group. In this group, melatonin (Sigma-Aldrich, MO, USA; catalogue number: M5250) was intraperitoneally administered (10 mg/kg) at 0.5 h after LPS injection, and then injected once daily until postnatal day 7. The vehicle for lipopolysaccharides was PBS; for melatonin, DMSO diluted with PBS was used. The number of rats for the respective experiments is shown in Table 1.

**Table 1:** Number of rats killed at various time points after the LPS intraperitoneal injection (inside the round brackets) or LPS + melatonin intraperitoneal injection (inside the square brackets) and their age-matched controls for different experiments (outside the brackets)

Age	Immunofluorescence	Western blotting	In situ hybridization	Electron microscopy
6 h	0	5 (5) [5]	0	0
1 day	5 (5) [5]	5 (5) [5]	0	0
3 days	5 (5) [5]	5 (5) [5]	0	0
7 days	5 (5) [5]	5 (5) [5]	0	0
14 days	5 (5) [5]	5 (5) [5]	5 (5) [5]	0
28 days	5 (5) [5]	5 (5) [5]	5 (5) [5]	5 (5) [5]

The survival and body weight of the rats were closely monitored after drug treatment. Rats were killed at different time points at 6 h, 1, 3, 7, 14 and 28 days following PBS/LPS/melatonin administration. All animal experimental procedures were consented by Institutional Animal Care and Use Committee, Guangdong Province, China. All efforts were made to reduce the number of rats used for experimentation and their suffering.

### Animal Behavior Test

#### Morris Water Maze Task

Morris water maze task was used to assess the learning and memory function of rats at P28d after LPS/melatonin injection. The Morris water maze device used in this study was composed of a circular water tank with a diameter of 200 cm. The tank was divided into four quadrants and filled with 30 cm deep water. The water temperature was kept at 25.0 ± 1.0 °C. A platform fixed in the target quadrant with a diameter of 9 cm, and a height of 29 cm, and an automatic camera tracking device. The surrounding environment remained consistent throughout the experiment. The steps of the Morris water maze task included adaptive swimming, spatial learning and space exploration. Adaptive swimming: on the first day of the task, the rats were permitted to swim freely for 120 s in the tank where the underwater fixed platform was removed. This was to ensure that each rat had the same amount of time to get acclimatized to the spatial information of the surrounding environment. Spatial learning: for the next 4 days, the rats were given a swimming test from the four quadrants at a fixed time each day, once per day. The rats entered the water with their heads up and facing the tank wall, and the time for the rats to arrive the fixed platform was known as the escape latency. If the rats could not reach the underwater platform within 120 s, its escape latency was recognized as 120 s, and then the rats were guided to the fixed platform underwater to take a rest for 60 s. Space exploration: on the last day (6th day) of this task, the underwater platform was removed before the test, and the rats entered the water from new start points. The numbers of times the rats passed the original platform position in 120 s were recorded.

#### Open Field Test

To exam exploratory activity in rats, the open field test was applied at P28d after LPS/melatonin injection. The open field device was a box consisting of a 75 × 75 cm plywood arena surrounded by 50 cm high walls. On the day of the formal test, rats were delivered to the testing room and allowed to leave from their home cages for 1 h before the test. Rats in each group were released in the center of the device and then left to explore the arena for 5 min. The distances moved and times spent in the center arena were recorded using Ethovision video tracking. The test lasted for 3 days. All animals were subjected to the open field once a day. The animals in three groups were tested in turn so that each group could be tested at the same period of time. The apparatus was thoroughly sterilized with 70% ethanol before the commencement of test for each animal.

#### Rotarod Test

Motor coordination and equilibrium were tested on a rotarod test of rats at P28d after LPS/melatonin injection. Each rat received a training course on the rotarod set at a constant speed of 30 rpm once a day for 3 days before the formal test. Rats can adapt the rotarod well after training. The test was terminated when the rats fell down the rod, and the time until they dropped from the rod was recorded. Three trials were carried out with 30-min intervals. For analysis, we calculated the average latency from the 3 trials performed for each rat. The number of experimental rats in each group in behavioral test is shown in Table 2.

**Table 2:** Number of rats in different groups in behavioral test at aged of 28 days

Group	Morris water maze task	Open field test	Rotarod test
Control	10	10	10
LPS injection	10	10	10
LPS + MT injection	10	10	10

### Primary Culture of Microglia

1-day-old SD rats were used for culture of primary microglia. Before the experiment, Poly-l-lysine (PLL, 0.1 mg/ml) was used for pre-cultured coating in a flask. The 1-day-old rats were disinfected with alcohol; after decapitation, the brain was quickly removed. With the aid of a dissecting microscope, the covering meninges were removed with a pair of fine forceps. Following this, the cerebral cortex was carefully dissected. The cerebral cortex was trimmed and minced into approximately 1 mm^3^-sized tissue blocks. The tissues were digested with 0.125% trypsin in a 37 °C water bath for 10 min. The digestion was terminated with FBS. The single-cell suspensions were allowed to settle for 5 min, and then filtered through a 200-mesh filter. The filtered single-cell suspension was centrifuged at 1000r/min for 5 min. In the next step, the supernatant fluid was discarded, and the mixed cells were incubated and cultured in DMEM/F12(Gibco) medium containing 10% fetal bovine serum (Hyclone, Logan, UT), and planted in a pre-PLL cultured 75 cm^2^ flask at a density of 1.2 × 10^6^ cells/mL. After 24 h, half of the medium was replaced with fresh medium, a procedure that was repeated every other day for 7–8 days. On the 8–9th day, the primary microglia were isolated and purified by constant temperature shaking table oscillations described in our previous study [46]. The specific steps were as follows: the culture bottle was placed on a shaking table at 37 °C for 1 h, with a rotating speed of 180 r/min; meanwhile, the primary microglia with poor surface adhesion force of the mixed glia cells were removed. Next, the purified microglia cells were collected and then fixed it on a 6-well petri dish precoated with PLL for further processing of subsequent experiments. Microglia identification was confirmed with Iba1 (Abcam, ab178846), a recognized marker for microglia; DAPI (Sigma-Aldrich, D9542) used for nucleus staining; only cultures showing over 98% purity of microglia were used in this study (Supporting Fig. 5A, B).

### Cell Counting Kit-8 Assay

To examine primary microglia viability induced by melatonin, a concentration gradient of melatonin at 0, 0.1, 0.5, 1, 2, 3 mM was used to treat the cells. Microglial cells were cultured in a 96-well plate. After adherence on the culture surface for 12 h, microglial cells were treated with different concentrations of melatonin and incubated for 24 h. Each well was then added with 10 μl of cell counting kit-8 (CCK-8) reaction reagent and incubated for 2 h. The absorbance was measured via the Microplate Reader according to the manufacturer’s manual.

### Treatment of Primary Microglia with LPS and Melatonin

Primary microglia were cultured in a medium containing DMEM/F12 (Gibco) and 10% fetal bovine serum (FBS) (Hyclone, Logan, UT) in a humidified incubator containing 5% CO_2_ and 95% air at 37 °C. For drug administration, primary microglia were pretreated with melatonin (1 mM) for 1 h and then incubated with medium containing 1 μg/ml LPS for 24 h. Primary microglia subjected to different treatments were divided into five different groups. Group I: To determine the optimal concentration of melatonin in reducing inflammation response. The cultured spheres of primary microglia were treated with different concentrations of melatonin. Microglial cells were assigned to five groups, including the control group (0.01 M PBS), LPS (1 μg/ml) group, LPS (1 μg/ml) + 0.5 mM melatonin group, LPS (1 μg/ml) + 1 mM melatonin group and LPS (1 μg/ml) + 2 mM melatonin group. Group II: To study the effect of LPS and melatonin on the expression of inflammatory cytokines in primary microglia. The cultured spheres of primary microglia were divided into three groups, including the control group (0.01 M PBS), LPS (1 μg/ml) group and LPS (1 μg/ml) + 1 mM melatonin group. The primary microglia in Group II were processed for immunocytochemical staining following the standard procedure. Group III: To investigate the mechanism of melatonin receptor-mediated anti-inflammatory effects in microglia. The cultured spheres of primary microglia were assigned to five groups, including the control group (0.01 M PBS), LPS (1 μg/ml) group, LPS (1 μg/ml) + 1 mM melatonin group, LPS (1 μg/ml) + 1 mM melatonin + 100 μM luzindole group [luzindole (the blocker of both MT1 and MT2 melatonin membrane receptors, abcam, ab145232) was added 1 h prior to melatonin treatment], and 1 mM melatonin group. Group IV: To examine whether the JAK2 pathway is involved in inflammatory response in microglia. The cultured spheres of primary microglia were assigned to four groups, including the control group (0.01 M PBS), LPS (1 μg/ml) group, LPS (1 μg/ml) + 1 mM melatonin group, LPS (1 μg/ml) + 1 mM melatonin + 10 μM AG490 group [AG490 (the blocker of JAK2, MCE, CAS No.133550-30-5) was added 1 h prior to melatonin treatment]. Group V: To verify if STAT3 pathway is involved in inflammatory response in microglial cells. The cultured spheres of primary microglia were divided into four groups, including the control group (0.01 M PBS), LPS (1 μg/ml) group, LPS (1 μg/ml) + 1 mM melatonin group, LPS (1 μg/ml) + 1 mM melatonin + 10 μM STAT-IN-3 group [STAT-IN-3 (the blocker of STAT3, MCE, CAS No.2361304-26-7) was added 1 h prior to melatonin treatment]. Group VI: To confirm the telomerase-mediated anti-inflammatory effects in microglia. The cultured spheres of primary microglia were assigned to five groups, including the control group (0.01 M PBS), LPS (1 μg/ml) group, LPS (1 μg/ml) + 1 mM melatonin group, LPS (1 μg/ml) + 1 mM melatonin + 10 μM BIBR 1532 group [BIBR 1532 (the blocker of telomerase reverse transcriptase, MCE, CAS No:321674-73-1) was added 1 h prior to melatonin treatment], and 1 mM melatonin group.

### Western Blot Analysis

The proteins from primary microglia with different treatments as well as fresh corpus callosum tissues were extracted using a protein extraction kit (Best Bio, BB-3101-100T). The bicinchoninic acid (BCA) method was used to detect the protein concentrations by BCA Protein Assay Kit (Thermo Scientific, 23250). Samples of supernatants containing 30 µg of total protein were heated to 100 °C for 10 min. Standard western blot procedures were performed as described in our previous study [3, 30, 46]. The primary antibodies and dilution concentration used in western blot are listed in Table 3. After three washes in tris-buffered saline Tween (TBST), the membranes were hybridized with the appropriate secondary antibodies, such as anti-mouse IgG (1:3000, Cell Signaling Technology, 7076S) or anti-rabbit IgG (1:3000, Cell Signaling Technology, 7074S) for 1 h at room temperature or 2 h at 4 °C. The protein bands were visualized by chemiluminescence kit (Millipore, WBKLS0500) and images were created by ImageQuant LAS 500 Imager (GE Healthcare Bio-Sciences AB). The optical density of the respective protein bands was quantified with image J software.

**Table 3:** Primary antibodies used in experiments

Antibody	Host	Company	Cat. No.	Application (Concentration)
IL-1β	Rabbit	Abcam	ab9722	WB (1:1000)/IF (1:200)
TNF-α	Rabbit	Abcam	ab66579	WB (1:1000)/IF (1:200)
iNOS	Rabbit	Novus	NB300-605	WB (1:1000)
TGF-β	Rabbit	Abcam	ab215715	WB (1:1000)
CD206	Rabbit	Abcam	ab64693	WB (1:1000)/IF (1:100)
TERT	Mouse	Santa Cruz Biotechnology	sc-377511	WB (1:1000)/IF (1:100)
MT1	Rabbit	Bioss	bs-0027R	WB (1:1000)/IF (1:100)
P-JAK2	Rabbit	Cell Signaling Technology	3776	WB (1:1000)
JAK2	Rabbit	Cell Signaling Technology	3230	WB (1:1000)
p-STAT3	Rabbit	Cell Signaling Technology	9145	WB (1:1000)
STAT3	Rabbit	Cell Signaling Technology	12640	WB (1:1000)
CNPase	Rabbit	Cell Signaling Technology	5664	WB (1:1000)/IF (1:200)
MBP	Mouse	Abcam	ab62631	WB (1:1000)
PLP	Rabbit	Abcam	ab28486	WB (1:1000)
NG2	Mouse	Abcam	Ab5009	WB (1:1000)/IF (1:200)
NFH	Rabbit	Abcam	ab8135	WB (1:2000)
NFM	Mouse	Abcam	ab7794	WB (1:1000)
NFL	Mouse	Cell Signaling Technology	2835s	WB (1:1000)/IF (1:200)
β-actin	Mouse	Cell Signaling Technology	3700s	WB (1:3000)
GAPDH	Rabbit	Abcam	ab181602	WB (1:3000)

### Electron Microscopy and G-Ratio Analysis

Blocks of corpus callosum tissue no more than 1 mm^3^ were freshly removed from P28 rats under a dissecting microscope. Care was taken not to cause any undesirable physical damage to the tissue during the removal. Tissue blocks were immediately fixed in a mixed aldehyde solution composed of 3% glutaraldehyde and 2% paraformaldehyde in 0.1 M PBS for 2–4 h for transmission electron microscopy (Servicebio, code: G1102) at 4 °C. After washing three times in 0.1 M PBS, the tissue was post-fixed with 1% OsO_4_ in 0.1 M PBS (pH 7.4) at room temperature for 2 h. The tissue blocks were embedded in an Epon mixture by baking in a 60 °C oven for 48 h. Ultrathin sections (60–80 nm) were cut on a ultramicrotome (Leica model: Leica UC7). The sections were stained with uranyl acetate in pure ethanol for 15 min, rinsed with distilled water, and then poststained with lead citrate for 15 min. Ultrathin sections were scrutinized using a transmission electron microscope (HITACHI, model:HT7700). Electron microscopic images were captured at 4000× and 12,000× magnification. Six non-overlapping electron microscope images were stochastically selected from each group to analyze the number of myelinated axons; the axon diameter and myelin sheath diameter were then measured by Image J software. G-ratio of the myelinated axon was then computed, that is, the ratio of the axon diameter to the axon plus the myelin sheath diameter. The data were analyzed by GraphPad software.

### Double Immunofluorescence

Brain tissues fixed in 4% paraformaldehyde and then kept in 30% sucrose were prepared. Frozen sections at 10-μm thickness were cut on a cryostat frozen microtome and mounted on slides. For double immunofluorescence staining, solution mixed with 5% BSA and 0.1% Triton X-100 in PBS was applied to block the frozen sections for 1 h at room temperature. Brain sections obtained from each time points in LPS group, LPS + melatonin group and their aged matching control group were assigned to five different groups. In group I: the brain sections derived from the corpus callosum of rats in each group sacrificed at 1 day after LPS intraperitoneal injection, LPS + melatonin intraperitoneal injection and their aged matching controls. They were incubated with IL-1β antibody. In group II: the brain sections derived from the corpus callosum of rats in each group sacrificed at 1 day after LPS intraperitoneal injection, LPS + melatonin intraperitoneal injection and their aged matching controls. They were incubated with CD206 antibody. In group III: the brain sections came from the corpus callosum of rats in each group sacrificed at 1 day after LPS intraperitoneal injection, LPS + melatonin intraperitoneal injection and their aged matching controls. They were incubated with MT1 antibody or TERT antibody. In group IV: the brain sections were from the corpus callosum of rats in each group sacrificed at 14 and 28 days after LPS intraperitoneal injection, LPS + melatonin intraperitoneal injection and their aged matching controls. They were incubated with antibody directed against anti-CNPase. In group V: the brain sections were from the corpus callosum of rats in each group sacrificed at 7, 14 and 28 days after LPS intraperitoneal injection, LPS + melatonin intraperitoneal injection and their aged matching controls. They were incubated with antibody directed against anti-NG2. In group VI: the brain sections were from the corpus callosum of rats in each group sacrificed at 7, 14 and 28 days after LPS intraperitoneal injection, LPS + melatonin intraperitoneal injection and their aged matching controls. They were incubated with antibody directed against anti-NFL. All the brain sections incubated with primary antibodies and dilution concentration used in double immunofluorescence staining (Table 3) were carried out overnight at 4 °C. After washing in PBS, the sections were incubated at room temperature for 1 h or 4 °C for 2 h with lectin or their corresponding fluorescent secondary antibodies. After three washes with PBS, DAPI (Sigma-Aldrich, D9542) was used for nucleus staining. The sections were viewed under a fluorescence microscope. The overall status immunofluorescence staining image of the corpus callosum is shown in Supplemental Fig. 1A (white box). The lesion area of the corpus callosum examined is shown in Supplemental Fig. 1A (red box).

The cultured spheres of primary microglia were administrated with PBS, LPS or LPS + melatonin for 24 h. After treatment, immunocytochemical staining was carried out in primary microglia following standard protocol. The primary microglia were incubated with primary antibodies against TNF-α or CD206 (Table 3) overnight at 4 °C. Next, the primary microglia were incubated with lectin and appropriate secondary fluorescent antibodies. Finally, the primary microglia were counterstained with DAPI.

After staining, the brain sections or cells were examined and images captured using a fluorescence microscope. For cell count, 5 frozen sections from each group were selected. For each section, five different microscopic fields in the corpus callosum or microglial cells at 40× magnification were scrutinized. The positive cell count was performed using Image J software. IL-1β+/lectin+/DAPI+, CD206+/lectin+/DAPI+, NG2+/DAPI+ or CNPase+/DAPI+ labeled cells were counted and recorded for statistical analysis.

### In Situ Hybridization

We carried out in situ hybridization on 15-μm-thick coronal frozen brain section from P14d and P28d rats. The protocol of in situ hybridization followed that described in our previous studies [3, 30]. After binding of horseradish peroxidase (HRP)-labeled probes, the brain sections were colored with chromogenic substrate, dehydrated with ethanol (2 min for 70% and 95%, separately), cleared with xylene for 5 min, and mounted. The probe information of PLP and MBP was described in our previous study [46]. PLP and MBP positive cells appeared as a distinct spot of chromogen precipitate visible through a common bright field microscope (Olympus Company, Japan) at 40 × magnification.

The overall status in situ hybridization image of the corpus callosum is shown in Supplemental Fig. 1B (white box). The lesion area of the corpus callosum analyzed in this study is shown in Supplemental Fig. 1B (red box). For each section, a minimum of five randomly selected microscope fields were scrutinized for enumeration of MBP and PLP-positive cells.

### Statistical Analysis

**Fig. 1:**
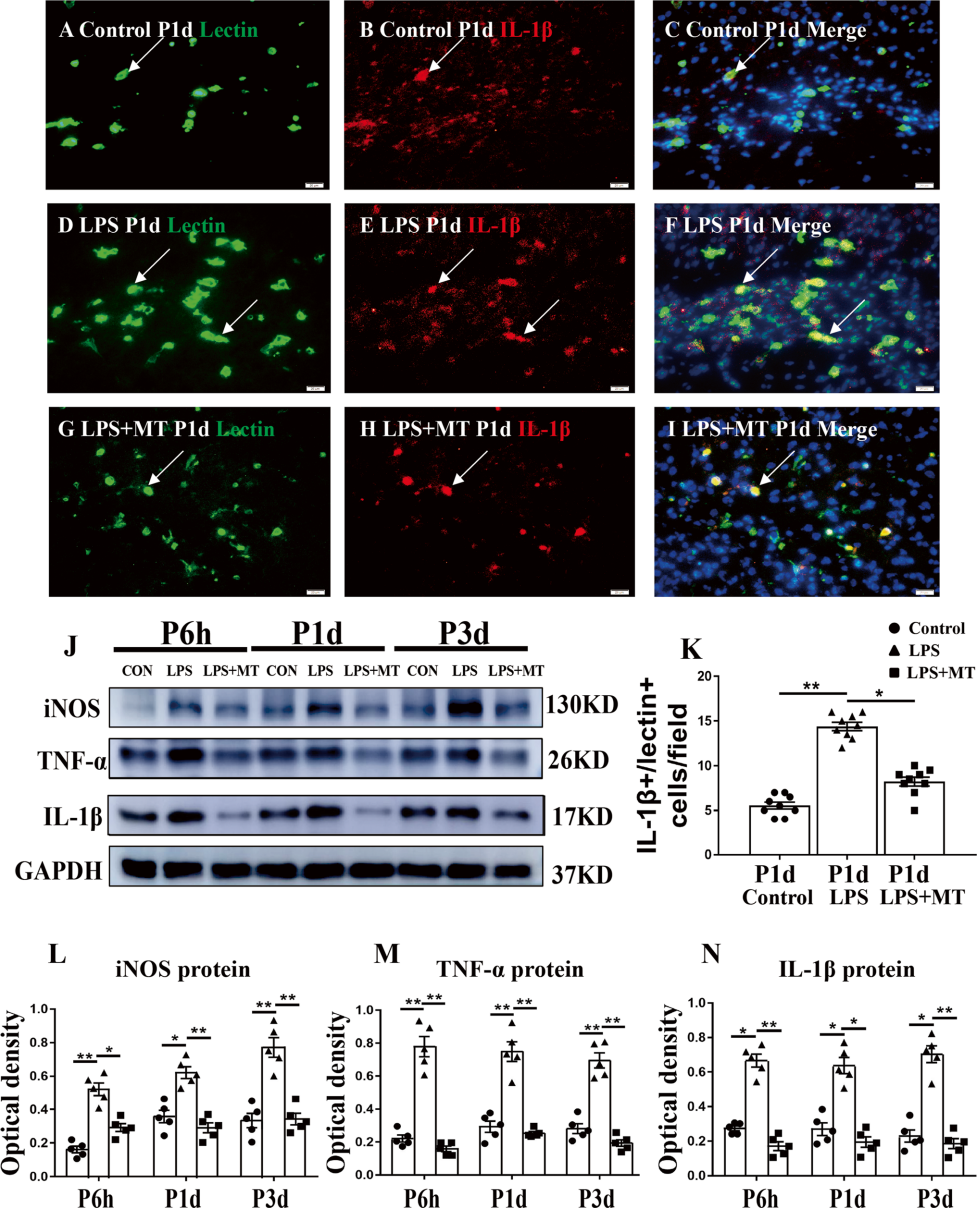
IL-1β, TNF-α and iNOS protein expression in the corpus callosum of postnatal rats at 6 h, 1 day and 3 days after LPS/melatonin injection and their matching controls. Double immunofluorescence staining shows the distribution of lectin labeled (**A**, **D**, **G** green), IL-1β (**B**, **E**, **H** red) and DAPI (blue) immunoreactive microglial cells in the corpus callosum at 1 day after the LPS/melatonin injection and their matching controls. The co-localized expression of lectin and IL-1β in microglia could be seen in **C**, **F** and **I**. IL-1β expression in microglia was increased at 1 day after LPS injection (**D**–**F**) when compared with control (**A**–**C**). However, IL-1β expression in microglia at 1 day was decreased after LPS + melatonin injection (**G**–**I**). Bar graph in **K** summarizes the frequency of IL-1β^+^/lectin^+^ cells at 1 day (*n* = 5 for each group). Quantification by immunoblot (**J**) showed increased IL-1β, TNF-α and iNOS protein expression at 6 h, 1 day and 3 days after LPS injection when compared with controls, but it was noticeably decreased in LPS + melatonin group. Graphs **L**, **M** and **N** show optical density changes of IL-1β, TNF-α and iNOS, respectively, relative to GAPDH (*n* = 5 for each group). In graph **K**–**N**, the circle, triangle and square represent the control groups, LPS groups and LPS + MT groups, respectively. Scale bars: **A**–**I** 20 µm. *P < 0.05, **P < 0.01

**Fig. 2:**
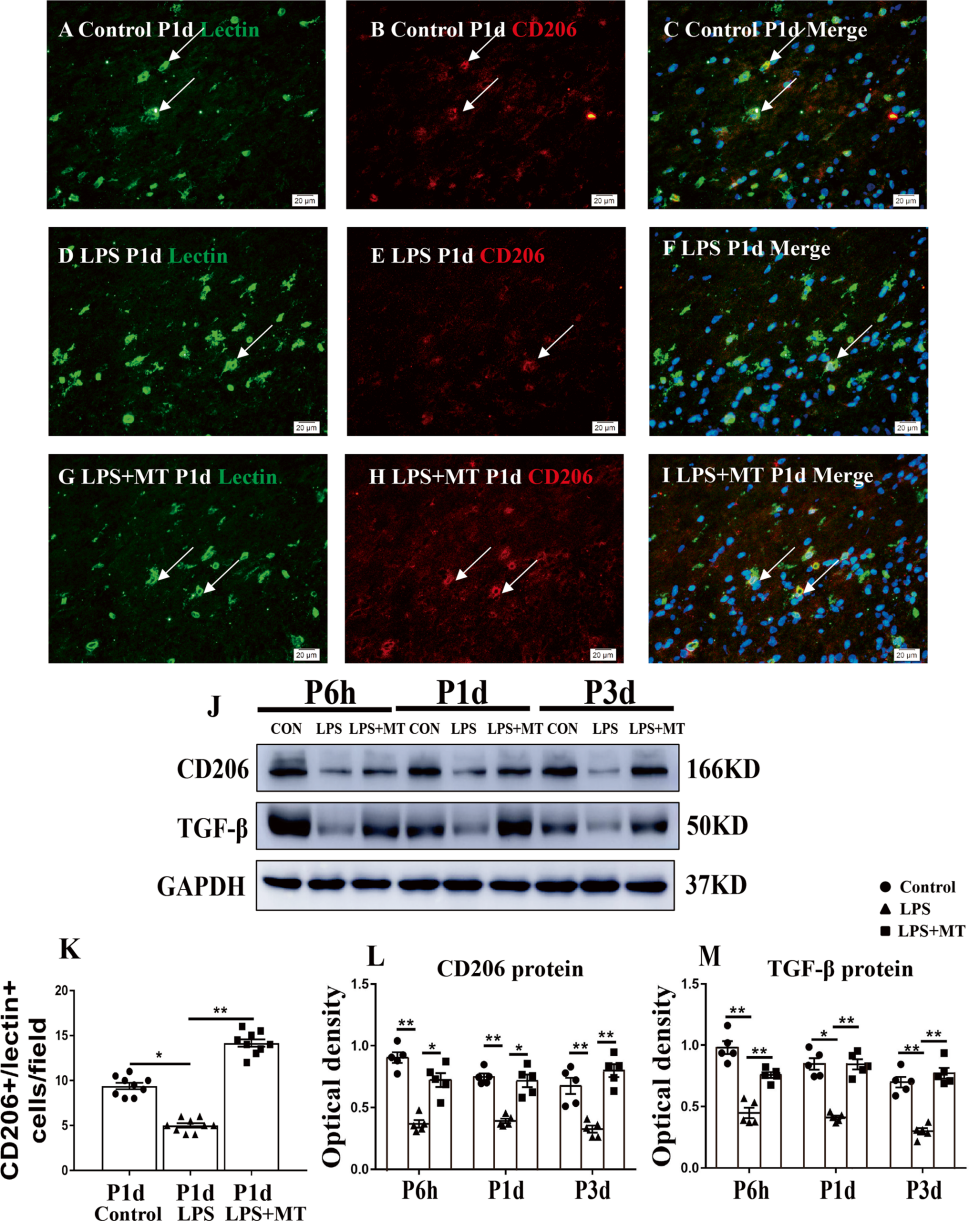
CD206 and TGF-β protein expression in the corpus callosum of postnatal rats at 6 h, 1 day and 3 days after LPS/melatonin injection and their matching controls. Double immunofluorescence staining shows the distribution of lectin labeled (**A**, **D**, **G** green), CD206 (**B**, **E**, **H** red), and DAPI (blue) immunoreactive microglial cells in the corpus callosum at 1 day after the LPS/melatonin injection and their matching controls. Co-localized expression of lectin and CD206 in microglia could be seen in **C**, **F** and **I**. Note CD206 expression in microglia was decreased at 1 day after LPS injection (**D**–**F**) when compared with control (**A**–**C**). However, CD206 expression in microglia at 1 day was increased after LPS + melatonin injection (**G**–**I**). Bar graph in **K** summarizes the frequency of CD206^+^/lectin^+^ cells at 1 day (*n* = 5 for each group). Quantification by immunoblot (**J**) showed decreased CD206 and TGF-β protein expression at 6 h, 1 day and 3 days after LPS injection when compared with controls, but it was noticeably increased in LPS + melatonin group. Graphs **L** and **M** show optical density changes of CD206 and TGF-β, respectively, relative to β-actin (*n* = 5 for each group). In graph **K**–**M**, the circle, triangle and square represent the control groups, LPS groups and LPS + MT groups, respectively. Scale bars: **A**–**I** 20 µm. *P < 0.05, **P < 0.01

**Fig. 3:**
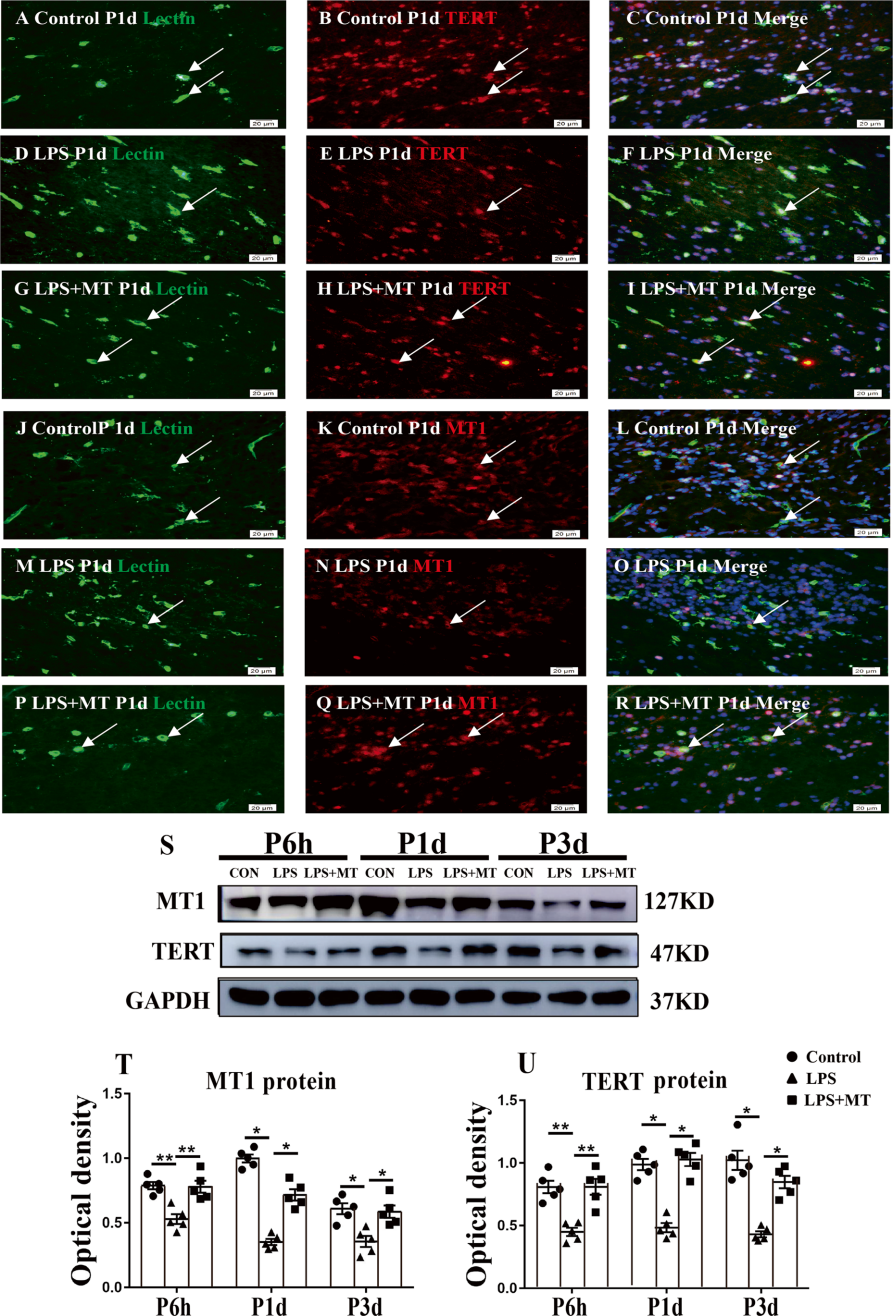
Protein expression of TERT and MT1 in the corpus callosum at 6 h, 1 day and 3 days of postnatal rats after LPS/melatonin administration and their matching controls. Panels **A**–**R** show lectin labeled and TERT or MT1 immunoreactive microglial cells in the corpus callosum at 1 day after LPS injection (**B**, **E**, **H**, **K**, **N**, **Q**), LPS + melatonin administration (**C**, **F**, **I**, **L**, **O**, **R**) and their corresponding controls (**A**, **D**, **G**, **J**, **M**, **P**), (*n* = 5 for each group). Graph **S**–**U** are western blot analysis of TERT and MT1 protein expression in the corpus callosum at 6 h, 1 day and 3 days of postnatal rats after LPS/melatonin administration and their corresponding controls. Graph **T** and **U** showed optical density changes of TERT and MT1 relative to GAPDH (*n* = 5 for each group). In graph **T**–**U**, the circle, triangle and square represent the control groups, LPS groups and LPS + MT groups, respectively. Note TERT and MT1 expression in microglia was attenuated at 1 day after LPS injection; but the expression of both proteins was increased after LPS + melatonin administration. Scale bars: **A**–**L** 20 µm. *P < 0.05, **P < 0.01

**Fig. 4:**
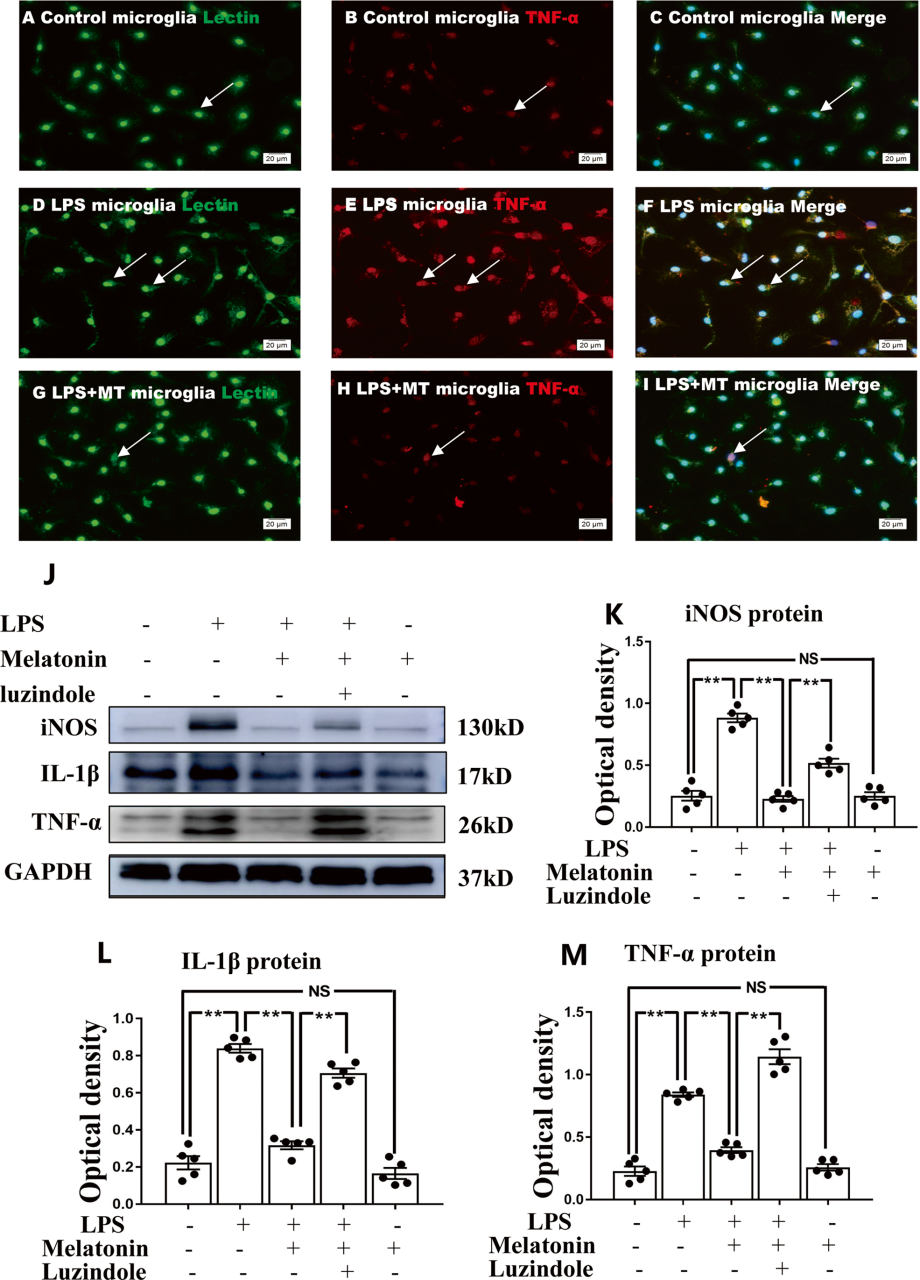
Melatonin reversed the enhanced expression of proinflammatory mediators in primary microglia stimulated by LPS in vitro. Immunofluorescence images of cultured primary microglia show expression of lectin (green), TNF-α(red) and DAPI (blue) (**A**–**I**) at 24 h after the LPS or melatonin treatment when compared with the corresponding control. Panel **J** shows iNOS (130 kDa), TNF-α (26 kDa), IL-1β (17 kDa) and GAPDH (37 kDa) immunoreactive bands. Bar graphs in **K**–**M** show optical density changes of iNOS, TNF-α and IL-1β relative to GAPDH of each group. Note LPS increased the expression of iNOS, TNF-α and IL-1β protein expression in primary microglia. Melatonin treatment could significantly reverse the high expression of IL-1β, iNOS and TNF-α proteins induced by LPS. Remarkably, the effect of melatonin was blocked by luzindole. Scale bars: **A**–**I** 20 µm. *P < 0.05, *n* = 5 for each group

**Fig. 5:**
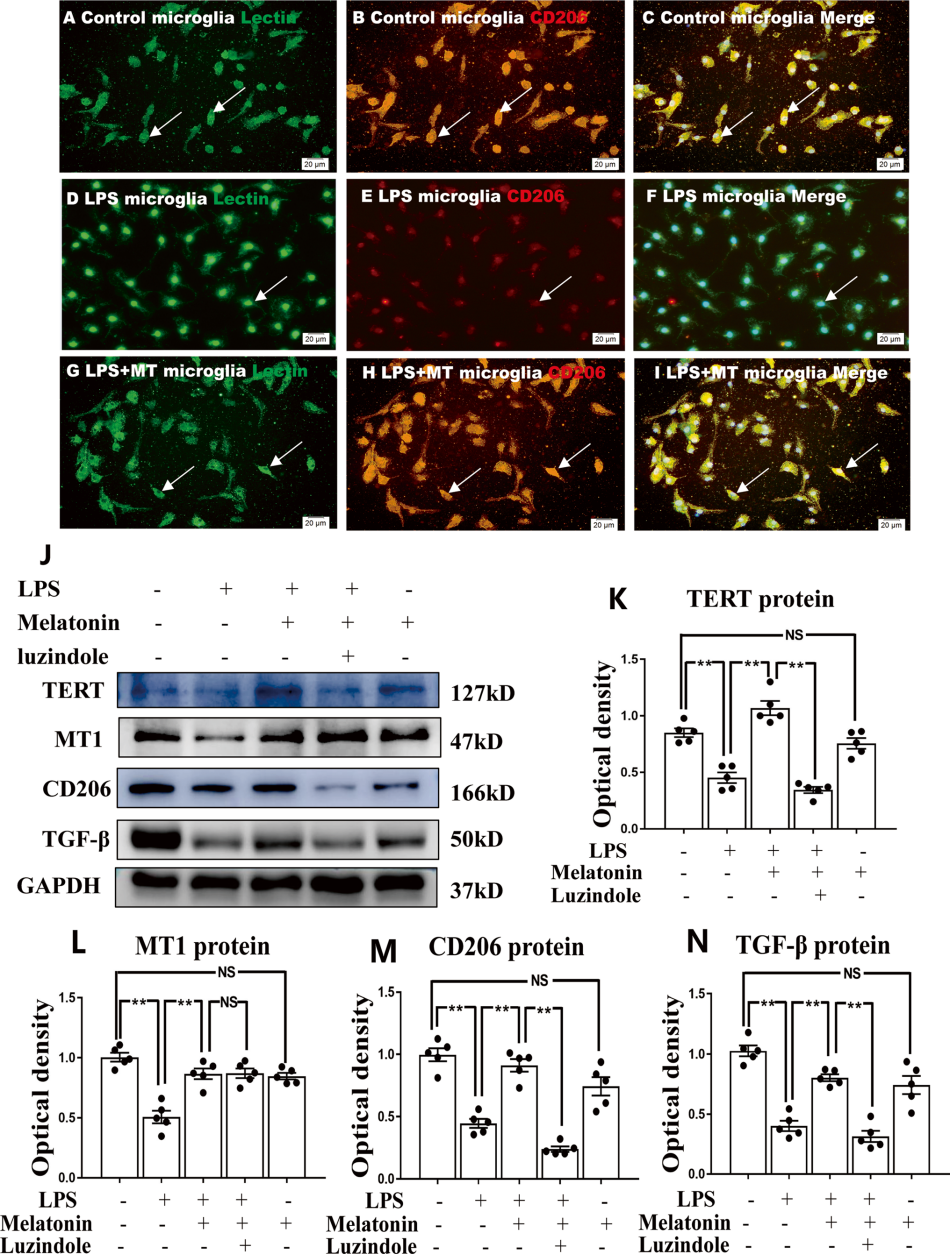
Melatonin reversed the low expression of anti-inflammatory mediators, TERT and melatonin receptor MT1 in primary microglia stimulated by LPS in vitro. Immunofluorescence images of cultured primary microglia show expression of lectin (green), CD206 (red) and DAPI (blue) (**A**–**I**) at 24 h after the LPS or melatonin treatment when compared with the corresponding control. Panel **J** shows CD206 (166 kDa), TGF-β (50 kDa), TERT (127 kDa), MT1 (47 kDa) and GAPDH (37 kDa) immunoreactive bands. Bar graphs in **K**-**N** show optical density changes of CD206, TGF-β, TERT, MT1 relative to GAPDH. Note LPS decreased the expression of CD206, TGF-β, TERT and MT1 protein expression in primary microglia. Melatonin treatment could significantly reverse the low expression of CD206, TGF-β, TERT and MT1 proteins induced by LPS. Of note, the effect of melatonin was blocked by luzindole. Scale bars: **A**–**I** 20 µm. *P < 0.05, *n* = 5 for each group

**Fig. 6:**
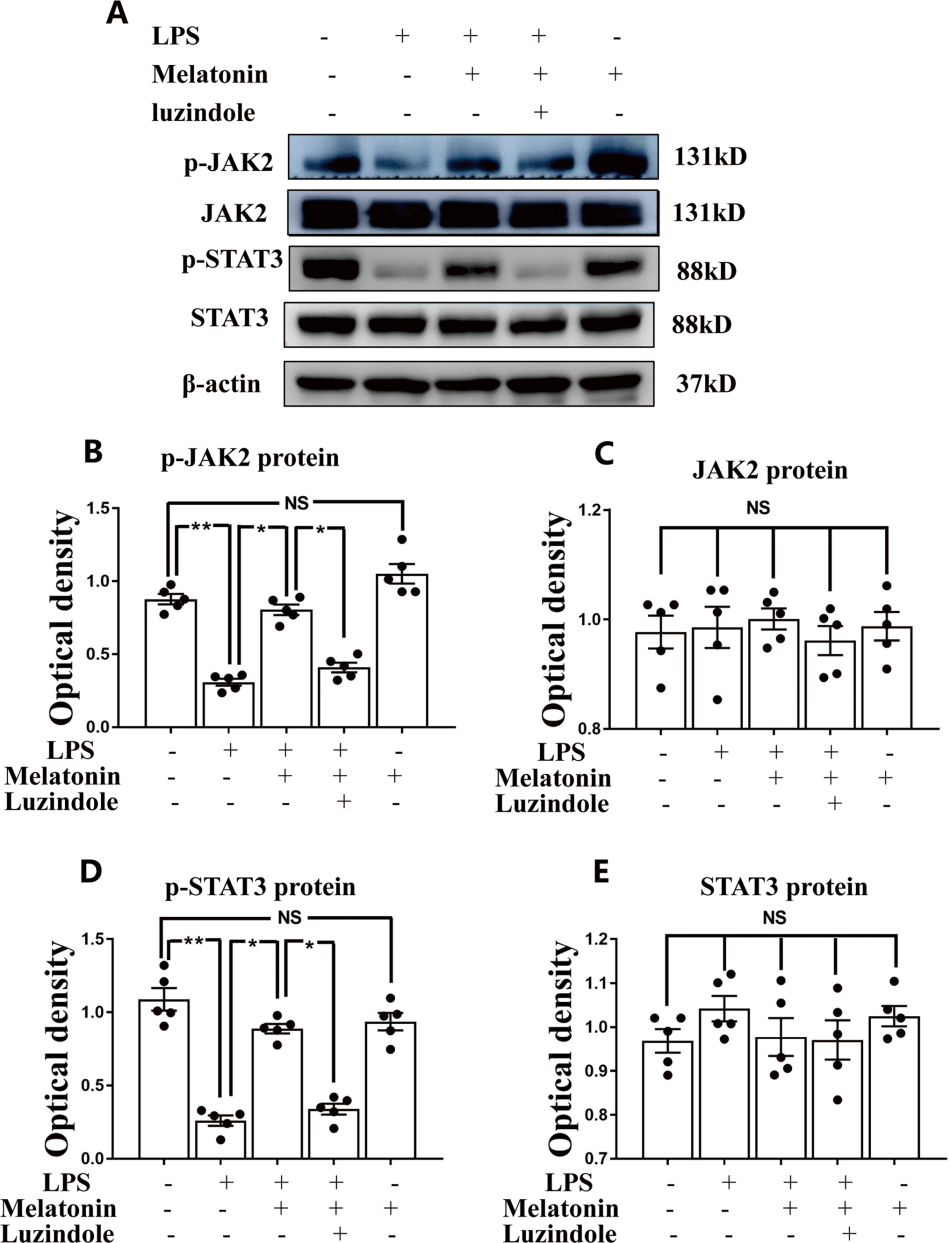
Melatonin activates JAK2/STAT3 pathway in microglia exposed to LPS in vitro. Panel **A** shows p-JAK2(131 kDa), JAK2(131 kDa), p-STAT3(88 kDa), STAT3(88 kDa) and β-actin(42 kDa) immunoreactive bands. Bar graphs in **B**-**E** show optical density changes of p-JAK2, JAK2, p-STAT3, STAT3 relative to β-actin of each group. Note melatonin treatment activates JAK2/STAT3 pathway and then modulate the conversion of M1 to M2 polarization. Note the effect of melatonin was blocked by luzindole. Scale bars: **A**–**I** 20 µm. *P < 0.05, *n* = 5 for each group

**Fig. 7:**
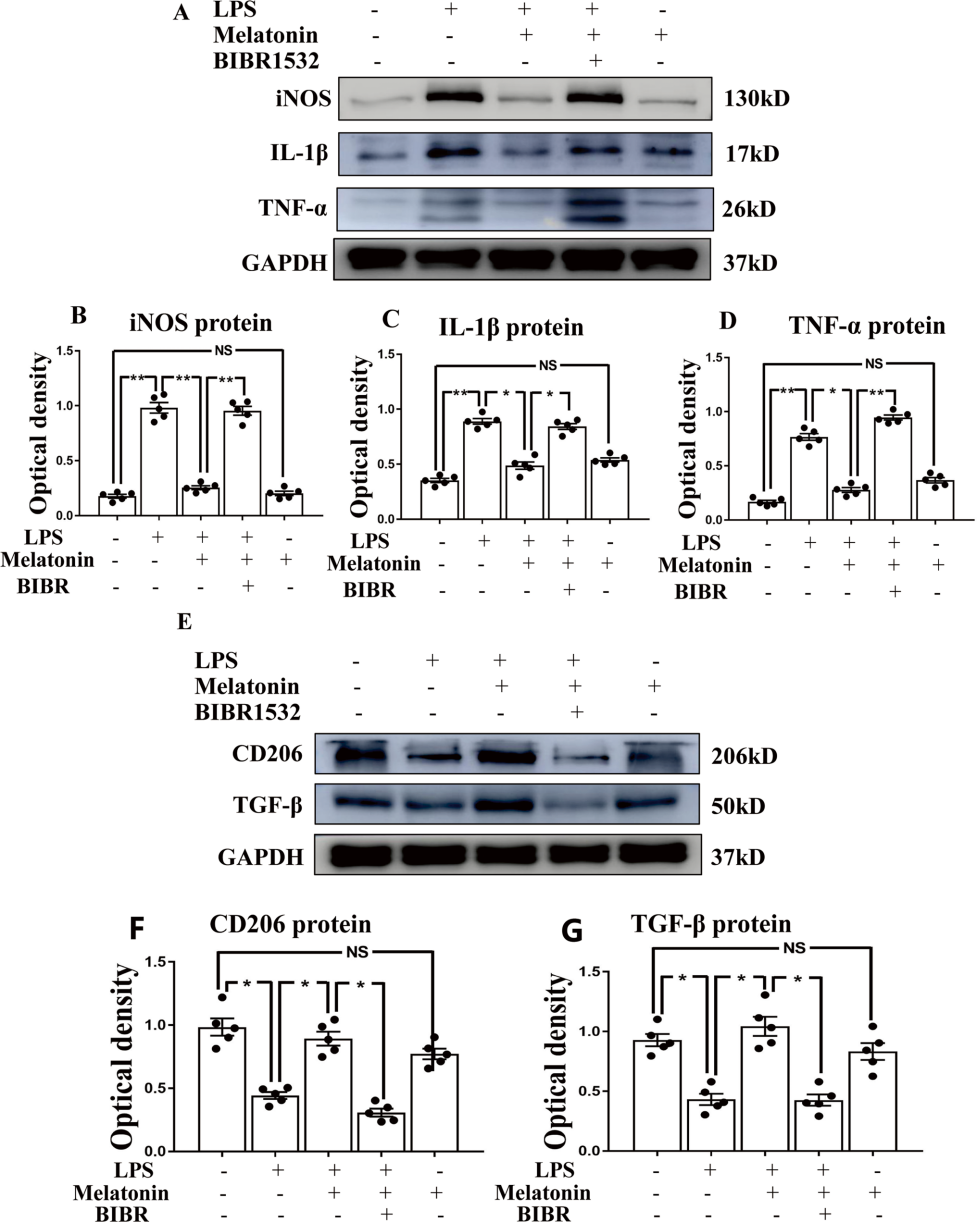
Melatonin modulates microglia polarization from M1 to M2 phenotype through increased telomerase expression in vitro. Panel **A** shows proinflammatory mediators, including iNOS (130 kDa), TNF-α (26 kDa), IL-1β (17 kDa) and GAPDH (37 kDa) immunoreactive bands and panel **E** shows anti-inflammatory mediators, including CD206 (166 kDa), TGF-β (50 kDa) and GAPDH (37 kDa) immunoreactive bands after the LPS, melatonin or BIBR (a non-competitive inhibitor of telomerase activity) treatment when compared with the corresponding control in primary microglia. Bar graphs in **B**–**D** and **F**–**G** show optical density changes of iNOS, TNF-α, IL-1β, CD206 and TGF-β relative to GAPDH of each group. Note melatonin treatment could significantly reverse the high expression of pro-inflammation proteins and also the low expression of anti-inflammation proteins induced by LPS. The protective effect of melatonin, however, was abrogated when telomerase expression was inhibited by BIBR1532. Scale bars: *P < 0.05, *n* = 5 for each group

All data are showed as the mean ± SEM. Statistical analyses were performed with IBM SPSS 20.0 statistical Software (USA). Different types of data obtained were subjected to the most suitable statistical method. The data in supporting Fig. 2A were analyzed by Chi-square test. The data in Figs. 1, 2, 3 and supporting Fig. 2B, 3, 4, 6, 7 variance with time (days after LPS injection) and treatment group (vehicle, LPS or melatonin) were analyzed using two-way ANOVA. The data in supporting Fig. 5 variance with axon diameter and treatment group (vehicle, LPS or melatonin) was analyzed using two-way ANOVA. The univariate-factor data in Figs. 4, 5, 6, 7 and supporting Fig. 2F–K, 8, 9, and 10 were analyzed using one-way ANOVA on account of the data were homogeneity of variance. P < 0.05 was considered as statistically significant.

## Results

### Behavioral Tests

#### Survival Rate and Body Weight of Rats

As shown in supporting Fig. 2A, the survival rate of rats decreased to 59% in the LPS group; however, it increased to 73% in the LPS + melatonin treatment group. A drastic decrease in body weight appeared to be one of the main effects of LPS injection in the first postnatal week; thereafter, the body weight was progressively increased with time in all groups. From the second week onwards, the body weight in both LPS and control animals increased significantly and was comparable in both groups. Of note, the body weight between the control and LPS + melatonin groups was not significantly different (Supporting Fig. 2B), suggesting that melatonin improved the survival rate as well as body weight of postnatal rats following LPS injection, and that it had no deleterious effects.

#### Memory/Cognitive Function Test by Morris Water Maze Task

At 28 days post drug treatment, the LPS-injected group exhibited a slow learning process as measured by the escape latency to find the fixed platform on day 3, 4, and 5; it was markedly prolonged in comparison with the control group. However, in LPS-injected group given melatonin treatment, a significant decrease in latency time was observed (Supporting Fig. 2F). Similarly, on the 6th day during the space exploration, the numbers of original platform crossings in the LPS group were markedly decreased compared with the control group. However, this was significantly improved in the LPS + melatonin group which showed an increase in numbers of platform crossings (Supporting Fig. 2G). It is therefore suggested that melatonin can improve memory performance in rats receiving LPS injection.

#### A Motor Balance and Coordination Assessment by the Rotarod Test

In LPS-injected group rats, the motor performance was drastically declined when compared with the control group. As shown in Supporting Fig. 2H, the LPS-injected rats consistently fell off the rod earlier than the control rats. In the LPS + melatonin group, rats remained on the rod for a longer duration than the LPS-injected group. The results suggest that melatonin can improve the motor balance and coordination of rats following LPS injection.

#### Open Field Test for Assessment of Locomotor Activities and Emotionality of Animals

As shown in Supporting Fig. 2I–J, in LPS-injected group, the rats moved for a shorter distance and spent a shorter duration in the center area than the control group. On the other hand, in LPS + melatonin group, the distance traveled and time spent in the center arena on the open field were longer than the LPS group. In consideration of the effect of LPS and melatonin on locomotion in terms of distance traveled by the rats, we also analyzed the ratio between the distance in the center arena and the total distance (Supporting Fig. 2K). We found that the ratio in LPS-injected group was markedly downregulated in comparison with the control group; but the ratio was increased in LPS + melatonin treatment group. Thus, the results suggest that melatonin can prevent the rats from emotionality alterations induced by LPS.

The above data obtained from behavioral studies showed that all the performances of LPS-injected rats in terms of spatial learning and memory, motor coordination and emotionality alterations were improved with melatonin treatment.

### IL-1β, TNF-α and iNOS Protein Expression in Corpus Callosum

It is well documented that microglia in cell colonies are preferentially distributed in the corpus callosum above the lateral ventricles in the developing brain [46]. On activation induced by LPS or hypoxia, microglia release excess amount of proinflammatory cytokines such as IL-1β and TNF-α that promote inflammatory response and aggravate tissue injury [15]. In view of this, this study focuses on microglia in the corpus callosum which constitutes a part of the periventricular white matter (PWM). Microglia were identified by lectin labeling in control, LPS and LPS + melatonin groups. In this study, we have used lectin to label microglia because it gives a better photo-image resolution for closer analysis as compared with Iba-1. As shown in Fig. 1, lectin^+^ cells were markedly increased in the corpus callosum in the LPS group at 1 day, but were decreased in LPS + melatonin group (Fig. 1A, D, G, K). Immunofluorescence labeling showed that IL-1β was weakly expressed in microglia in control group. IL-1β immunofluorescence, however, was obviously enhanced in the LPS group. In LPS + melatonin group, IL-1β immunofluorescence in microglia was noticeably attenuated when compared with that in LPS group (Fig. 1A–I, K). The area of corpus callosum analyzed is shown in Supporting Fig. 1A. The immunoreactive bands of IL-1β, TNF-α and iNOS protein levels in the corpus callosum that appeared at approximately 17 kDa, 26 kDa and 130 kDa, respectively, showed increased optical density at 6 h, 1 day and 3 days after LPS injection; but it was decreased in LPS + melatonin group (Fig. 1J, L–N). The results indicated that melatonin can inhibit microglia activation in the corpus callosum and reduce the production of proinflammatory cytokines by them in LPS-injected rats. This is evident by the diminution of IL-1β immunofluorescence in microglia notably at 1 day; additionally, the protein levels of IL-1β, TNF-α and iNOS were downregulated at 6 h, 1 day and 3 days in LPS-injected rats with melatonin treatment.

### CD206 and TGF-β Protein Expression in Corpus Callosum

It is evident from the above that microglia which accumulated in the corpus callosum showed increased expression of proinflammatory cytokines, namely, IL-1β and TNF-α in LPS-injected group. Both cytokines are considered to be the characteristic biomarkers for M1 microglia phenotype. We next investigated if they also expressed M2 phenotype markers i.e., CD206 and TGF-β that promote inflammation resolution and tissue repair. As shown in Fig. 2 lectin^+^ microglia increased in numbers in the corpus callosum in the LPS group at 1 day, but were decreased in LPS + melatonin group (Fig. 2A, D, G). CD206 immunofluorescence was intensely expressed in microglia in control group. In LPS group, CD206 immunofluorescence in microglia was noticeably decreased. However, in LPS + melatonin group, CD206 immunofluorescence in microglia was evidently augmented when compared with that in LPS group (Fig. 2A–I, K). The area of corpus callosum analyzed is shown in Supporting Fig. 1A. By western blotting, the immunoreactive bands of CD206 and TGF-β protein levels in the corpus callosum that appeared at approximately 166 kDa and 50 kDa, respectively, showed decreased optical density at 6 h, 1 day and 3 days after LPS injection; the optical density, however, was increased in LPS + melatonin group (Fig. 2J, L–M). The results indicated that melatonin can modulate microglia polarization towards the M2 phenotype and reduce generation of proinflammatory cytokines in LPS-injected rats. This is evident by the decrease in IL-1β but increase in CD206 immunofluorescence intensity microglia noticeably at 1 day in LPS + melatonin rats. Further support was gained from the western blot analysis which showed that the protein levels of IL-1β, TNF-α and iNOS were downregulated, whereas that of CD206 and TGF-β was upregulated at 6 h, 1 day and 3 days in LPS-injected rats given melatonin treatment.

### TERT and MT1 Protein Expression in Corpus Callosum

We next investigated the role of telomerase and melatonin receptor MT1 in microglia in the corpus callosum in LPS-injected rats treated with melatonin. Double labeling showed that TERT and MT1 immunofluorescence was co-localized in lectin labeled microglia at 1 day in the control rats (Fig. 3A–C, J–L). Both TERT and MT1 immunofluorescence in microglia was attenuated after LPS injection, but was evidently enhanced in LPS-injected rats given melatonin treatment (Fig. 3A–I, J–R). The area of corpus callosum analyzed is shown in Supporting Fig. 1A. By western blot, the immunoreactive bands of TERT and MT1 protein expression in the callosal tissue at approximately 127 kDa and 47 kDa showed a marked decrease in optical density at 6 h, 1 and 3 days after LPS injection as compared with their age-matching controls; of note, the expression of both proteins was significantly upregulated in LPS-injected rats receiving melatonin treatment (Fig. 3S–U). The results showed that LPS injection could reduce TERT and MT1 expression in activated microglia in the corpus callosum and that melatonin treatment could revert the decreased expression level of both proteins after LPS injection.

### Melatonin Attenuated Axonal Hypomyelination in the Corpus Callosum

We reported previously wide occurrence of hypomyelination affecting many axons in the corpus callosum in postnatal rats at 14 and 28 days after LPS injection [3, 46]. Here we have focused our study on determining whether melatonin treatment would reverse the long-term impairments on myelination caused by LPS. First, we investigated the expression of myelin-associated proteins including PLP, MBP and CNPase which are specific markers for mature myelination protein in the CNS. Immunofluorescence showed that CNPase protein expression was reduced in the corpus callosum in postnatal rats sacrificed at 14 and 28 days after LPS injection, and appeared to recover in the LPS + melatonin group at 14 and 28 days (Supporting Fig. 3A–F); likewise, the frequency of CNPase + cells was restored in the latter group (Supporting Fig. 3H). The area of corpus callosum analyzed is shown in Supporting Fig. 1A. The optical density of immunoreactive bands of PLP, MBP and CNPase protein expression levels in the corpus callosum was significantly decreased at 14 and 28 days after LPS administration (Supporting Fig. 3G, I–K). Very strikingly, melatonin treatment reversed the decreased expression of all three myelin proteins affected by LPS injection (Supporting Fig. 3G, I–K).

To verify the above, we examined the MBP^+^ and PLP^+^ oligodendrocytes through in situ hybridization with antisense riboprobes targeted at MBP and PLP. The area of corpus callosum analyzed is shown in Supporting Fig. 1B. The results showed that melatonin reversed the decline of MBP^+^ and PLP^+^ oligodendrocytes in the corpus callosum at 14 and 28 days after LPS injection (Supporting Fig. 4A–L, N–O).

We next analyzed the axonal myelination and myelin thickness in the corpus callosum by electron microscopy to ascertain if they underwent pathological changes. Electron microscopic images of axonal profiles at 4000× and 12,000× showed that the myelinated axons in the corpus callosum were more sparsely distributed in the LPS group at 28 days. In the LPS group, the myelin sheaths often appeared thinner, and in some instances, disrupted or disorganized (Supporting Fig. 5B, E) when compared with the matching control (Supporting Fig. 5A, D). Melatonin treatment resulted in an increase in the frequency of myelinated axons. Moreover, hypomyelination caused by LPS injection became less evident (Supporting Fig. 5C, F). The ratio of axon diameter to axon diameter plus myelin sheath thickness is defined as the g-ratio, which is independent of axon diameter and reliable data for evaluating myelination. The g-ratio was measured for myelinated axons in different groups in the corpus callosum. The average g-ratios of the callosal axons in the LPS-induced rats were markedly increased compared with their matching control group (Supporting Fig. 5G). In axons of different diameters ranging from 0.2 to 1.2 μm based on 12,000× image, thinner myelin was found in the LPS injection group. Melatonin improved the myelination of axons after LPS injection (Supporting Fig. 5H). When taken together, the results indicate that melatonin can promote myelin synthesis in the corpus callosum in LPS-injected rats as evident by not only the increased myelin protein expression but also by the increase in myelin thickness and structural integrity.

### Melatonin Rescues Differentiation and Maturation of Oligodendrocytes in the Corpus Callosum in LPS-Injected Rats

To investigate the effect of melatonin on OPC development, we used NG2 (a marker for OPCs) to evaluate the maturation of OPCs in the corpus callosum. Immunofluorescence labeling showed that the number of NG2^+^ OPCs was significantly increased at 7, 14 and 28 days following LPS injection (Supporting Fig. 6B, E, H) in comparison with the age-matched controls (Supporting Fig. 6A, D, G). Melatonin treatment resulted in decline in number of NG2^+^ OPCs (Supporting Fig. 6C, F, I). Thus, a noticeable increase in number of NG2^+^ cells was observed in the corpus callosum at 7, 14 and 28 days (Supporting Fig. 6L) after LPS injection. However, the incidence of NG2^+^ cells was significantly decreased at 7, 14 and 28 days after melatonin treatment (Supporting Fig. 6L). The area of corpus callosum analyzed is shown in Supporting Fig. 1A. The immunoreactive band of NG2 protein that appeared at approximately 260 kDa showed decreased optical density at 7, 14 and 28 days after melatonin treatment compared with the LPS groups (Supporting Fig. 6J, K). The results suggest that the decline in myelin proteins expression in the corpus callosum at 7, 14 and 28 days was attributed to reduced numbers of mature oligodendrocytes. It is noteworthy that melatonin significantly diminishes the suppression effect of LPS on the differentiation and maturation of OPCs.

### Neurofilament Protein Expression in Corpus Callosum

We reported previously that the expression of neurofilament proteins NFL, NFM, and NFH played an important role in the axonal caliber of myelinated axons and that it was significantly reduced in LPS-injected rats [3]. Here, we further explored the effect of melatonin on the expression of neurofilament proteins in LPS-injected postnatal rats. As shown in Supporting Fig. 7J–M and by Western blot analysis, NFH, NFM, and NFL protein expression was drastically downregulated in the corpus callosum at 7, 14, and 28 days after LPS injection when compared with their age-matching controls. However, melatonin treatment countered the decline in NFH, NFM, and NFL protein expression following LPS injection (Supporting Fig. 7J–M). Immunofluorescence labeling showed that NFL expression in the corpus callosum was evidently reduced at 7, 14, and 28 days following LPS injection (Supporting Fig. 7B, E, H) when compared with the age-matched controls (Supporting Fig. 7A, D, G). Very strikingly, melatonin treatment could reverse the decrease in NFL expression induced by LPS injection (Supporting Fig. 7C, F, I). The area of corpus callosum analyzed is shown in Supporting Fig. 1A.

### Melatonin Modulates Microglia Polarization from M1 to M2 Phenotype Through JAK2-STAT3-Telomerase Pathway

The CCK-8 assay indicated that there was no statistical difference in viability of microglia subjected to different concentrations of melatonin treatment. Microglia, however, showed a moderate decline when the cells were treated with melatonin at the concentration of 3 mM (Supporting Fig. 8C). By western blot, it was further confirmed that melatonin at concentrations of 1 and 2 mM reduced the expression of proinflammatory mediators significantly (Supporting Fig. 8D–G). In view of this, melatonin at 1 mM was used as the working concentration to treat microglial cells. To further investigate whether melatonin would modulate microglia polarization from M1 to M2 phenotype, the protein expression levels of pro- and anti- inflammatory mediators in primary microglia in the control, LPS and LPS + melatonin group were compared. Immunofluorescence labeling showed that TNF-α, a M1 phenotype marker was weakly expressed (Fig. 4A–C), whereas CD206, the M2 phenotype marker was strongly expressed in the control primary microglia (Fig. 5A–C). However, the immunofluorescence intensity of TNF-α was markedly enhanced (Fig. 4D–F), whereas that of CD206 was weakly expressed at 1 day after LPS injection (Fig. 5D–F). It is noteworthy that the immunofluorescence intensity of both biomarkers was attenuated in cells subjected to melatonin pretreatment for 1 h before being challenged with LPS (Figs. 4G–I and 5G-I). Western blot analysis showed that the expression levels of IL-1β, iNOS and TNF-α proteins were significantly increased after LPS treatment when compared with the controls. Concomitant to this was a low expression of anti-inflammatory mediators of CD206 and TGF-β proteins (Figs. 4J–M and 5J, M, N). However, melatonin significantly reversed the low expression of CD206 and TGF-β and high expression of IL-1β, iNOS and TNF-α proteins induced by LPS (Figs. 4J–M and 5J, M, N). Remarkably, the effect of melatonin in modulating M1 to M2 phenotype was blocked by luzindole, a melatonin receptor inhibitor. It can be confidently concluded therefore that melatonin can reduce inflammatory response by shifting microglia from M1 to M2 phenotype and that it is melatonin receptor-dependent.

Recent studies have reported that TERT expression was localized in microglia and that it plays a protective role [39]. In light of this, we have extended the study to examine whether LPS and melatonin would affect TERT expression in microglia. Western blot analysis showed that TERT protein expression level was significantly decreased after LPS treatment when compared with the controls (Fig. 5J, K). However, melatonin significantly reversed the TERT diminution induced by LPS (Fig. 5J, K). Along with the above, p-JAK2 and p-STAT3 protein expression was significantly decreased after LPS treatment when compared with the controls in primary microglia (Fig. 6A, B, D). However, when treated with melatonin, the expression level of p-JAK2 and p-STAT3 was significantly elevated (Fig. 6A, B, D). The results suggest that the JAK2-STAT3 pathway might be involved in the process of melatonin modulating microglia polarization. To verify this, we used the inhibitors of JAK2 and STAT3 to treat primary microglia. Remarkably, the effect of melatonin in modulating M1 to M2 phenotype was blocked by AG490, a JAK2 inhibitor (Supporting Fig. 9A–J) and STAT-IN-3, a STAT3 inhibitor (Supporting Fig. 10A–H). However, when treated with JAK2 and STAT3 inhibitors the TERT expression level was significantly decreased when compared with the LPS + melatonin groups (Supporting Figs. 9E, F and 10E, F).

Arising from the above results, we conclude that melatonin can suppress the expression of proinflammatory mediators through upregulating TERT expression microglia. In this connection, we investigated whether BIBR 1532 (a non-competitive inhibitor of telomerase activity) would reverse the effect of melatonin in suppressing the high expression of proinflammatory mediators stimulated by LPS. Thus, by western blot, a significant increase in the protein expression level of IL-1β, iNOS and TNF-α (Fig. 7A–D) coupled with a decrease in the protein expression level of CD206 and TGF-β (Fig. 7E–G) was found after LPS + melatonin + BIBR treatment in comparison with the LPS + melatonin using western blot. These findings therefore suggested that melatonin acts through reducing the inflammatory response in microglia by modulating M1 to M2 polarization through JAK2-STAT3-telomerase pathway.

## Discussion

In the cerebrum, the PWM which includes the corpus callosum overlies the two lateral ventricles. This layer of white matter is particularly vulnerable to injury especially in premature infants. Some of the factors which have been reported to contribute to the PWMD include hypoxia, ischemia, inflammatory reactions and generation of free radicals [47, 48]. The pathogenesis of PWMD is complex and multifactorial. Damage to axons and immature oligodendrocytes prior to the onset of myelination has been described as a hallmark feature of PWMD [49]. We have reported previously a marked increase in production of a multitude of proinflammatory factors such as IL-1β, iNOS, IL-6 and TNF-α in the hippocampus and PWM in LPS-injected postnatal rats [3, 30, 46, 50]. Additionally, we have found that postnatal rats subjected to LPS treatment exhibited cognitive disorder at 28 days after LPS injection [51]. All this suggested that systemic LPS injection, which conceivably would have induced a clinical sepsis, had elicited robust inflammation in CNS and caused extensive brain damage affecting in particular the hippocampus and PWM, where innate immune cells microglia are known to preponderate [3, 30, 46, 52].

The vulnerability of PWM to inflammatory response mediated by activated microglia and the resulting myelination disorder played a major role in the pathogenesis of PWMD [13, 14]. Although many molecular mechanisms have been identified in brain dysfunction in septic postnatal rats, there are limited intervention strategies. Compared with the adult brain, the developing brain is considered to have a greater potential for regeneration after injury [48]. We therefore reasoned that promoting the differentiation of OPCs into mature oligodendrocytes such as the use of melatonin may contribute to the recovery of PWMD in septic postnatal rats. Although the antioxidant, anti-inflammation and anti-apoptotic properties of melatonin have been well documented in different animal models, the intrinsic ability of melatonin to promote differentiation and maturation of OPCs remains uncertain. Several experimental studies have emphasized the neuroprotective effects of melatonin when given as either a curative or prophylactic treatment in different experimental models of brain injury, such as Parkinson’s disease, Alzheimer’s disease and ischemic brain injury [25, 26, 53]. In adult melatonin-deficient rats, the lesions caused by hypoxia and ischemia were found to be larger than those in the control group, indicating that endogenous melatonin is neuroprotective [53]. In this connection, a promising strategy would be to reduce neuroinflammation with melatonin as well as to activate the maturation and differentiation of OPCs using a sepsis model as adopted in this study. The strategy may help promote brain repair and restoration of impaired brain functions. The present study was therefore undertaken to explore the underlying molecular and cellular mechanisms responsible for the neuroprotective effects of melatonin in LPS-induced white matter damage in the developing rat brain.

The present results have shown that melatonin effectively protects the brain against biochemical damage and behavioral alterations in the brain in postnatal rats with sepsis induced by LPS. Thus, melatonin-treated rats showed shorter escape latency and increased times of passing the original platform location when compared with the LPS-treated rats in the Morris water maze test. It is therefore suggested that melatonin can improve the cognitive function in postnatal sepsis rats. In the rotarod test, melatonin effectively improved the motor performance as compared with the LPS-injected rats without melatonin treatment. This was evident by the improvement in the duration spent on the rotating spindle as compared with the LPS-treated rats. Furthermore, in the open field test, melatonin increased the time and distance of visits to the center arena of the open field, indicating a decrease in anxiety in the animals. The behavioral tests systemically demonstrated the neuroprotective effects of melatonin in the rescue of dysfunction in the brain following LPS-induced sepsis in postnatal rat. It is relevant to note from recent studies that melatonin could improve the cognitive function of animals with chronic cerebral hypoperfusion, Alzheimer’s disease and injury induced by γ-hydroxybutyric acid intoxication [54–56]. The present results therefore are in accord with these studies and support that melatonin is beneficial for protection of neurological functions.

To further demonstrate the neuroprotective effects of melatonin in postnatal sepsis, we next investigated the underlying mechanism of melatonin that may be involved in improving neurological dysfunction as observed in the behavioral tests. First, and in agreement with previous studies, expression of proinflammatory mediators e.g. IL-1β, iNOS and TNF-α localized preferentially in microglia was drastically increased in the LPS-injected postnatal rats [30, 50]. In addition, we found that expression of anti-inflammatory mediators e.g. CD206 and TGF-β was decreased. Melatonin was found to attenuate the expression of proinflammatory mediators and improve the expression of anti-inflammatory mediators at the protein level. It is well documented that when challenged with appropriate stimuli, microglia polarize either towards a proinflammatory phenotype (M1) or an anti-inflammatory phenotype (M2). Decreased expression of proinflammatory mediators may be attributed to decreased M1 microglial polarization, while increased expression of anti-inflammatory mediators may be due to increase of M2 microglial polarization as was observed in the corpus callosum in LPS-injected rats receiving melatonin treatment. Several studies have reported in different perinatal brain injury models that immune cells are activated and that melatonin appeared to be intimately linked to inflammation modulation [24]. It would appear that some of the melatonin actions are receptor-mediated, while others are direct [28, 29]. Two major subtypes of melatonin receptor MT1 and MT2 have been localized in the brain cells including microglia, astrocytes, oligodendrocytes and neurons [57]. The present results have shown unequivocally that MT1 protein expression was decreased in the corpus callosum after LPS injection, but it was increased after melatonin treatment. In light of this, it is suggested that the neuroprotective effects of melatonin may be mediated by receptor-dependent pathways.

We reported previously that activated microglia play a major role in the early stage of inflammation cytokine generation, whereas astrocytes are involved in the release of inflammatory factors in the late stage of brain pathologies over a prolonged period in LPS-injected postnatal rats. The present study focused on the neuroprotective effects of melatonin. Importantly, we have shown that the neurohormone acts through modulating microglial polarization towards the M2 phenotype that is considered to be neuroprotective. Studies have indicated that melatonin receptor is highly expressed in microglia and astrocyte; it is weakly detected in oligodendrocytes [57]. It is therefore suggested that melatonin is likely to act directly on microglia as demonstrated in this study. Two possible explanations may be offered whereby melatonin can prevent axonal hypomyelination in the PWM in septic neonatal rats. Firstly, the PWM including the corpus callosum in postnatal rats is immature which is extremely vulnerable to ischemia, hypoxia and inflammation [58]. The present results have shown that melatonin can act directly through its receptor localized on microglia. The binding of melatonin with its receptor MT1 suppresses M1 microglial polarization and improves M2 microglial polarization resulting in attenuation of inflammatory response. In the latter, it is conceivable that the improved microenvironment with reduced proinflammatory mediators and increased anti-inflammatory mediators would help promote structural and functional recovery. Secondly, melatonin may act directly on oligodendrocytes bearing its cognate receptors and reduce cell damage. On the other hand, it has been reported that melatonin receptors are mainly expressed in microglia. It is therefore suggested that melatonin may act preferentially on microglia and modulate its polarization towards the M2 phenotype. M2 phenotype being anti-inflammatory would then help alleviate axonal hypomyelination in the PWM in septic postnatal rats [57].

The next issue to be addressed would be how the receptor-dependent pathway of melatonin might be involved in reducing inflammation in axonal hypomyelination in the white matter resulted from LPS administration. We next explored whether melatonin would reverse the disorder of axonal myelination resulted from LPS injection. The white matter in the CNS including the corpus callosum is mainly composed of closely packed myelinated axons. The formation of myelin is a complicated process in which oligodendrocytes interact with axons. In fact, the differentiation and maturation of OPCs, the complete development of axons, and the proper environment of the CNS are critical to the formation of myelin. Mature oligodendrocytes originate from OPCs, which have the ability to migrate, proliferate and differentiate into myelinated oligodendrocytes. We have shown in the present results that expression of PLP, CNPase and MBP, which are marker proteins of mature oligodendrocytes, was significantly upregulated while that of NG2, a marker protein of immature oligodendrocytes, was significantly decreased in the corpus callosum of LPS-injected rats after melatonin treatment. Ultrastructural studies confirmed that in LPS-injected rats given melatonin treatment, the frequency of myelinated axonal profiles was increased. Moreover, myelin sheath associated with the axons became thicker and appeared intact comparable to that in the control rats. It is suggested therefore that melatonin could revert the extensive hypomyelination in the white matter caused by LPS.

At the molecular level, axon development is known to be regulated by many specific proteins like NFL, NFM and NFH. There is evidence that low expression of these axon development associated proteins would affect the development of axon leading to its hypomyelination. By immunofluorescence and western blot analysis, we have shown that melatonin increases the expression of NFL, NLM and NFH in the corpus callosum in LPS-injected rats. Taken together, the present results indicate that melatonin could recover the disorder in differentiation and maturation of OPCs and increase the expression of some axon development associated protein in the corpus callosum of LPS-injected postnatal rats. In light of the above, it is concluded that melatonin can promote the differentiation and maturation of OPCs and attenuate axonal hypomyelination in LPS-injected rats.

To further ascertain whether melatonin would affect the polarization of microglia, primary microglia were treated with LPS, melatonin and luzindole (MT1 and MT2 melatonin receptor inhibitor); after this, expression of IL-1β, iNOS, TNF-α, CD206 and TGF-β in vitro was followed. Melatonin downregulates the increased expression level of IL-1β, iNOS and TNF-α; meanwhile, it upregulates the decreased expression level of CD206 and TGF-β in primary microglia stimulated by LPS. Remarkably, increased production of proinflammatory mediators and decreased expression of anti-inflammatory mediators in microglia by LPS was restored when melatonin receptor antagonist (luzindole) was added to melatonin treatment. Thus, it can be confidently concluded that melatonin reduces inflammation response stimulated by LPS through shifting microglial polarization towards the anti-inflammatory M2 phenotype in receptor-dependent manner.

Because melatonin can significantly shift the M1 microglia towards M2 phenotype, we next investigated the pathways involved in this polarization process. Accumulating evidence indicated that the JAK2/STAT3 plays a key role in cell proliferation, migration and immune cell maintenance [59]. In the present study, we found that the expression levels of p-JAK2 and p-STAT3 proteins in primary microglia were decreased after LPS treatment but they were noticeably increased when treated with melatonin. In view of this, we used AG490 (an inhibitor of JAK2) and STAT-IN-3 (an inhibitor of STAT3) to further confirm the involvement of JAK2/STAT3 pathway in the process of melatonin modulating the conversion of M1 microglia to M2. The results showed that when JAK2 was inhibited with AG-490 and STAT3 was inhibited with STAT-IN-3, the protective effects of melatonin against inflammation response were attenuated. This supports that JAK2/STAT3 pathway mediates the protective effects of melatonin in modulating the polarization of microglia.

The underlying neuroprotective mechanism of melatonin has remained obscure although it is well characterized for its endocrine, autocrine and paracrine actions. Additionally, it is endowed with anti-inflammatory and antioxidant properties. Relevant to the present discussion is TERT expression localized in neurons and microglia in vitro. Studies have shown that JAK/STAT signaling pathway is a positive regulator of TERT expression by direct binding of STAT3 or STAT5 to the TERT promoter. These findings indicate a close interplay between JAK2 signaling and telomerase or TERT. It has been reported that TERT expression is essential in the CNS development, and its expression is maintained into the adulthood in rodents [34]. A major finding in the present study was the significant decrease in TERT protein expression in the corpus callosum after LPS injection in postnatal rats; but it was upregulated after melatonin treatment. In primary microglia, TERT expression was decreased after LPS treatment, but it was upregulated following melatonin administration. In addition, we found that TERT protein expression was downregulated after luzindole, AG490 or STAT-IN-3 treatment. Very interestingly, adding BIBR1532 a telomerase activity inhibitor along with melatonin aggravated the inflammation response. The results suggest that the telomerase protein TERT is a protective factor in neural cells against inflammation and, in microglia, it acts to dampen the production of proinflammation mediators. In other words, the telomerase activators would be potential therapeutic agents for amelioration of neuropathological progression driven chiefly by activated microglia.

The present results have demonstrated unequivocally that melatonin administered intraperitoneally can help mitigate microglia-mediated neuroinflammation. More importantly, melatonin improves the delay of differentiation and maturation of OPCs including myelination. We have shown that the neuroprotective action of melatonin is achieved through its receptors and JAK2/STAT3/telomerase modulation in microglia. The finding, which we consider it to be novel, has amplified the neuroprotective role of melatonin. Most importantly, the underlying molecular mechanism of melatonin especially of its role in modulating conversion of microglia from M1 toward M2, a key cell player in postnatal sepsis, has been better clarified and deciphered.

## Conclusion

**Fig. 8:**
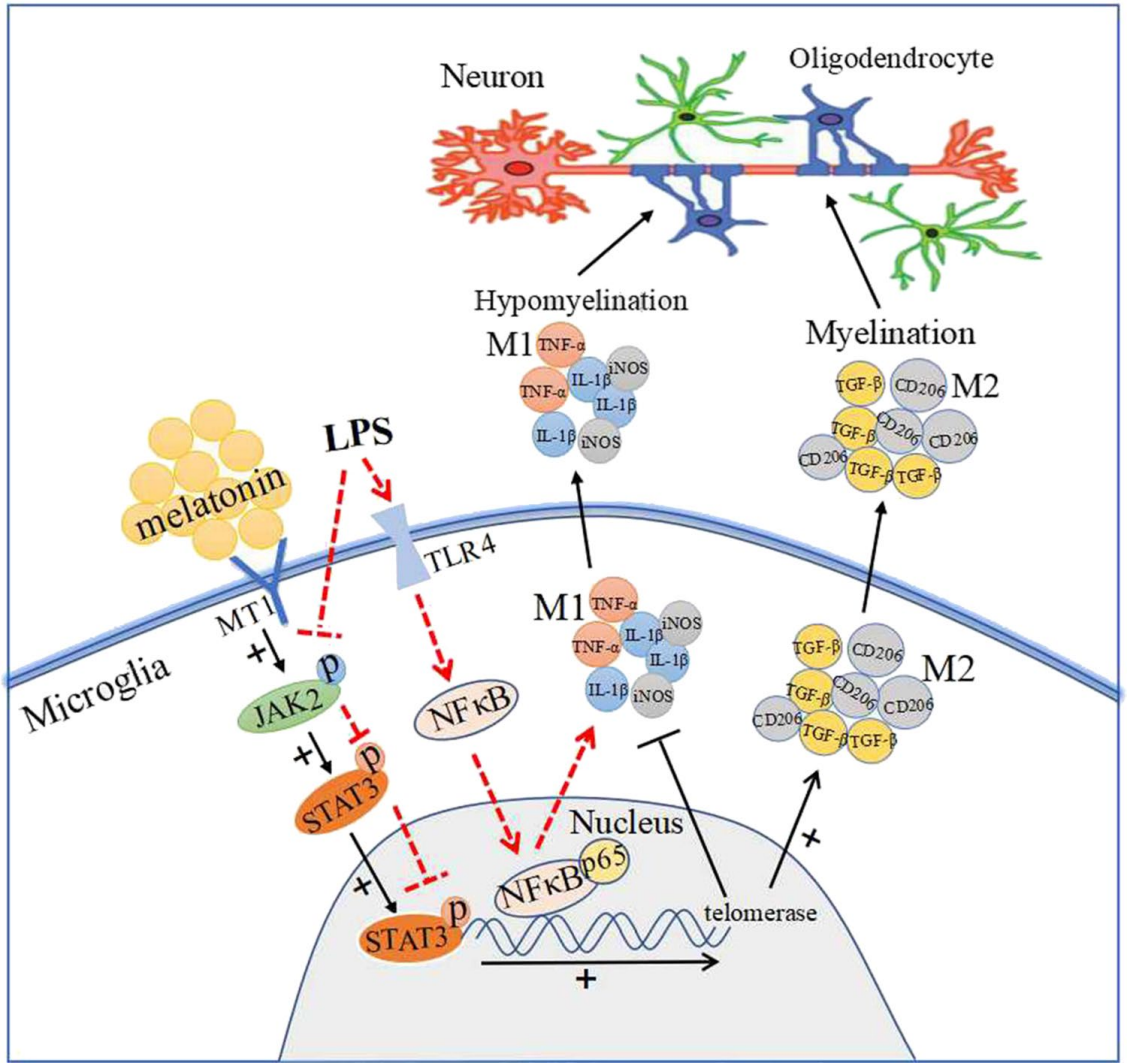
Table of Contents Image (TOCI): a schematic diagram depicting the cellular and molecular events associated with melatonin treatment in postnatal rats given LPS injection. The illustration follows two paths: the solid line shows that melatonin binds to its cognate receptor (MT1) on the microglia which activates the JAK2/STAT3 pathways and increases the expression of telomerase in the nucleus. This decreases M1 microglial production of proinflammatory cytokines, such as IL-1β, TNF-α and iNOS, and increases M2 production of anti-inflammatory cytokines, such as CD206 and TGF-β. Dash line denotes the effect of LPS on microglia. LPS stimulates toll-like receptor 4 (TLR4) which recruits its downstream NF-κB pathway, and ultimately mediates the production of proinflammatory mediators [59]. However, LPS inhibits the activation of JKA2/STAT3 pathway and reduces the expression p-JAK2 and p-STAT3 proteins expression, which is contrary to melatonin treatment. Production of proinflammatory cytokines by M1 microglia causes hypomyelination in the corpus callosum after LPS administration; while production of anti-inflammatory cytokines by M2 induced by melatonin improves hypomyelination

It is unambiguous from this study that melatonin can ameliorate neurobehavioral disturbances and alleviate axonal hypomyelination in the PWM of LPS-injected postnatal rats at 28 days. In this connection, melatonin increased the number of CNPase^+^, PLP^+^, MBP^+^ mature oligodendrocytes and decreased the number of NG2^+^ oligodendrocyte progenitor cells in the corpus callosum of postnatal rats following LPS administration. Furthermore, melatonin could reduce neuroinflammation and promote the conversion of M1 microglia phenotype to M2, as evident by the decrease of proinflammatory cytokines including TNF-α, IL-1β, iNOS and the augmentation of anti-inflammatory cytokines such as TGF-β and CD206. This was coupled with increased TERT and MT1 proteins expression. In vitro, the effect of melatonin on the conversion of M1 microglia towards M2 was modulated through JAK2/STAT3/telomerase pathway. All in all, it is suggested that melatonin could alleviate neuroinflammation and attenuate the axonal hypomyelination through modulating microglia polarization towards anti-inflammatory genotype M2 via JAK2/STAT3/telomerase pathway (Fig. 8). Arising from the present results, we conclude that melatonin may prove to be a potential therapeutic agent for converting M1 microglia into M2 phenotype, which would contribute to improving PWMD in LPS-injected postnatal rats.

## Supplementary Information

Below is the link to the electronic supplementary material.

Supporting Fig. 1 The overall status image of corpus callosum. A (immunofluorescence) and B (in situ hybridization) showed the area of the corpus callosum analyzed in this study. The white box shows the corpus callosum in brain. The red box shows the lesion area of the corpus callosum as analyzed in this study. Supplementary file1 (TIF 1460 kb)

Supporting Fig. 2 Melatonin improved behavioral performance in the LPS injected rat model. The survival rate (A) and body weight of rats (B) following LPS injection with or without melatonin treatment and their corresponding controls. (C-G) Cognitive performance in the Morris water maze task. The rats at 28d in LPS + melatonin group showed shorter escape latencies (F) and increased number of times crossing the original platform location (G) than the LPS group. (H)The rotarod test showed that melatonin treatment significantly improved the impairments in motor deficits of animals after LPS injection as evident by the longer duration staying on the rotarod. (I-K) The effects of melatonin on behaviors in the open field test. In LPS + melatonin group, the recorded time (I) and distance (J) in the center area were longer than the LPS group. In LPS + melatonin group the ratio between distance in the center arena and the total distance (K) was increased than the LPS group. *P<0.05, **P<0.01, n=10 for each group in every test. Supplementary file2 (TIF 31211 kb)

Supporting Fig. 3 Melatonin reversed the decline in expression of myelin associated proteins in the corpus callosum of postnatal rats at 14 and 28d after LPS injection. (A-F) CNPase immunofluorescence in the corpus callosum in postnatal rats at 14 and 28d after LPS (B, E) or LPS + melatonin (C, F) injection and their corresponding controls (A, D). Bar H summarized the frequency of CNPase+/DAPI+ cells at 14 and 28d after LPS/melatonin injection when compared with their corresponding controls (n=5 for each group). (G)Western blot analysis of PLP, MBP and CNPase protein expression levels in the corpus callosum of postnatal rats at 14 and 28d after LPS or LPS + melatonin injection and their matching controls. Graph I-K depicted the optical density changes of CNPase, PLP and MBP, respectively, relative to GAPDH, (n=5 for each group). It is evident that melatonin can reverse the decreased expression of CNPase, PLP and MBP protein in the corpus callosum induced by LPS exposure at 14 and 28d. Scale bars: A-F 20µm. *P<0.05, **P < 0.01. Supplementary file3 (TIF 37790 kb)

Supporting Fig. 4 Melatonin increased the cell numbers of PLP+ and MBP+ oligodendrocytes in the corpus callosum after LPS injection as revealed by in situ hybridization. (A-L) In situ hybridization shows the number of PLP+ and MBP+ oligodendrocytes in the corpus callosum at 14 and 28d after LPS injection (B, E, H, K), LPS + melatonin administration (C, F, I, L) and the corresponding controls (A, D, G, J) at the magnification of ×40. Note melatonin treatment reverses the decreased number of PLP+ and MBP+ oligodendrocytes in the corpus callosum induced by LPS exposure at 14 and 28d. Bar graph (M, N) summarized the frequency of PLP+ and MBP+ oligodendrocytes in the corpus callosum at 14 and 28d using in situ hybridization. Scale bars: A-L 20µm. *P < 0.05, **P < 0.01, n=5 for each group. Supplementary file4 (TIF 40700 kb)

Supporting Fig. 5 Melatonin prevented hypomyelination in the corpus callosum after LPS injection as revealed by electron microscopy. Electron microscopic images of corpus callosum at the magnification of ×4000 (A-C) and ×12000 (D-F). A-F show transverse section of myelinated axons in the corpus callosum at 28d after LPS (B, E) and LPS + melatonin injection (C, F) and their corresponding controls (A, D). Scatter diagram of g-ratio to axon diameter in the corpus callosum at 28d after LPS and LPS + melatonin injection and their matching controls were shown in bar graph G. H is bar graph showing g-ratio of myelinated axons of different diameters in the corpus callosum at 28d after LPS and LPS + melatonin injection and corresponding control. Scale bars: A-C 2 µm, D-F 500nm. *P < 0.05, **P < 0.01, n=5 for each group. Supplementary file5 (TIF 32291 kb)

Supporting Fig. 6 Effect of melatonin on the differentiation and maturation of OPCs in the corpus callosum of postnatal rats after LPS/melatonin injection. Immunofluorescence staining showing NG2 labelled oligodendrocytes (green) and DAPI (blue) in the corpus callosum in postnatal rats at 7, 14 and 28d after LPS (B, E, H) and LPS + melatonin injection (C, F, I) and their corresponding controls (A, D, G) at the magnification of ×40. (L) Bar graphs show the number of NG2+/DAPI+ in the corpus callosum at 7, 14 and 28d after LPS injection, LPS + melatonin administration and their matching controls (n=5 for each group). (J) Western blot analysis of NG2 expression levels in the corpus callosum in postnatal rats at 7, 14 and 28d after LPS/melatonin injection and their matching controls. Graph K showed optical density changes of NG2 relative to β-actin (n=5 for each group). Scale bars: 20 µm. *P < 0.05, **P < 0.01. Supplementary file6 (TIF 42352 kb)

Supporting Fig. 7 Melatonin upregulated the expression of neurofilament proteins NFL, NFM and NFH expression which played an important role in the axonal caliber of myelinated axons. Immunofluorescence staining shows NFL immunofluorescence (red) and DAPI (blue) in the corpus callosum in postnatal rats at 7, 14 and 28d after LPS (B, E, H) and LPS + melatonin injection (C, F, I) and their matching controls (A, D, G) at the magnification of ×40 (n=5 for each group). Panel J shows NFL, NFM, and NFH immunoreactive bands. (K-M) Bar graphs depict the optical density of NFL, NFM, and NFH expression shown in J. Quantification by immunoblot (J) shows significant decrease in NFL, NFM, and NFH protein expression at 7, 14 and 28d after LPS treatment in comparison with control; however, melatonin administration upregulates the expression of NFL, NFM, and NFH protein expression significantly (n=5 for each group). Scale bars: 20 μm. *P < 0.05, **P < 0.01. Supplementary file7 (TIF 44814 kb)

Supporting Fig. 8 Microglia purity test and the effect of different concentrations of melatonin on microglia viability and the optimal working concentration of melatonin. Iba1 was used as a marker for microglia, and DAPI was used for nucleus staining. Microglia cultures over 98% purity were used in this study. (A, B) CCK-8 assay showed microglia viability treated with a concentration gradient of melatonin at 0, 0.1, 0.5, 1, 2, 3 mM for 24h (graph A). Panel B shows pro-inflammatory mediators, including iNOS (130kDa), TNF-α (26kDa), IL-1β (17kDa) and GAPDH (37kDa) immunoreactive bands after the LPS and LPS + different concentrations of melatonin (0.5 mM, 1 mM and 2 mM) treatment when compared with the corresponding control in primary microglia. Bar graphs in B-E show optical density changes of iNOS, TNF-α and IL-1β relative to GAPDH of each group. For this study, 1mM melatonin was adopted as the optimal working concentration to treat microglia in different experiments. Scale bars: * P<0.05, n=5 for each group. Supplementary file8 (TIF 40668 kb)

Supporting Fig. 9. Melatonin modulates microglial polarization from M1 to M2 phenotype through JAK2/STAT3 pathway in vitro. Panel A shows pro-inflammatory mediators, including iNOS (130kDa), TNF-α (26kDa), IL-1β (17kDa) and GAPDH (37kDa) immunoreactive bands. Panel E shows anti-inflammatory mediators, including CD206 (166kDa), TGF-β (50kDa), TERT (127kDa) and GAPDH (37kDa). Panel I shows p-STAT3 (88kDa), STAT3 (88kDa) and GAPDH (37kDa) immunoreactive bands after LPS, melatonin or AG490 (an inhibitor of JAK2 activity) treatment when compared with the corresponding control in primary microglia. Bar graphs in B-D, F-H and J-K show optical density changes of iNOS, TNF-α, IL-1β, CD206, TGF-β, p-STAT3, STAT3 relative to GAPDH of each group. Note melatonin modulates the conversion of M1 to M2 polarization through JAK2/STAT3 pathway. Scale bars: * P<0.05, n=5 for each group. Supplementary file9 (TIF 38962 kb)

Supporting Fig. 10. Melatonin modulates microglial polarization from M1 to M2 phenotype through JAK2/STAT3 pathway in vitro. Panel A shows pro-inflammatory mediators, including iNOS (130kDa), TNF-α (26kDa), IL-1β (17kDa) and GAPDH (37kDa) immunoreactive bands and panel E shows anti-inflammatory mediators, including CD206 (166kDa), TGF-β (50kDa), TERT (127kDa) and GAPDH (37kDa) immunoreactive bands after the LPS, melatonin or STAT-IN-3 (an inhibitor of STAT3 activity) treatment when compared with the corresponding control in primary microglia. Bar graphs in B-D and F-H show optical density changes of iNOS, TNF-α, IL-1β, CD206 and TGF-β relative to GAPDH of each group. Note melatonin treatment modulates the conversion of M1 to M2 polarization through JAK2/STAT3 pathway. Scale bars: * P<0.05, n=5 for each group.Supplementary file10 (TIF 35281 kb)

## Data Availability

All data generated or analyzed during this study are included in this published article.

## Abbreviations

M1: A classically activated Microglial Phenotype 1
M2: An alternatively activated Microglial Phenotype 2
PWM: Periventricular White Matter
PWMD: Periventricular White Matter Damage
IL-1β: Interleukin-1β
TNF-α: Tumor Necrosis Factor-α
iNOS: Inducible Nitric Oxide Synthase
CD206: Macrophage Mannose Receptor
TGF-β: Transforming Growth Factor-β
MT: Melatonin
MT1: Melatonin Receptor 1
TERT: Telomerase Reverse Transcriptase
TERC: Telomerase RNA Component
LPS: Lipopolysaccharide
OPCs: Oligodendrocyte Progenitor Cells
JAK2: Janus Kinase 2
STAT3: Signal Transducer and Activator of Transcription
NG2: NG2 Proteoglycan
PLP: Proteolipid
MBP: Myelin Basic Protein
CNPase: 2′,3′-Cyclic Nucleotide 3′-Phosphodiesterase
NFH: Neurofilament-200
NFL: Neurofilament-68
NFM: Neurofilament-160
BCA: Bicinchonininc acid
BSA: Bovine Serum Albumin
CCK-8: Cell Counting Kit 8
CNS: Central Nervous System
DAPI: 4′6-Diamidino-2-Phenylindole
DMEM: Dulbecco’s Modified Eagle Medium
FBS: Fetal Bovine Serum
BBB: Blood–Brain-Barrier
PBS: Phosphate-Buffer Saline
NMDA: N-Methyl-D-Aspartate
TBS: Tris-Buffered Saline
TBST: Trisbuffered Saline Tween-20

## References

[1] Shane AL, Sanchez PJ, Stoll BJ (2017). Neonatal sepsis. Lancet.

[2] Zhao HQ, Li WM, Lu ZQ, Sheng ZY, Yao YM (2015). The growing spectrum of anti-inflammatory interleukins and their potential roles in the development of sepsis. J Interferon Cytokine Res.

[3] Xie D (2016). IL-1beta induces hypomyelination in the periventricular white matter through inhibition of oligodendrocyte progenitor cell maturation via FYN/MEK/ERK signaling pathway in septic neonatal rats. Glia.

[4] Rousset CI (2006). Maternal exposure to LPS induces hypomyelination in the internal capsule and programmed cell death in the deep gray matter in newborn rats. Pediatr Res.

[5] Wimalasundera N, Stevenson VL (2016). Cerebral palsy. Pract Neurol.

[6] Ahlin K (2013). Cerebral palsy and perinatal infection in children born at term. Obstet Gynecol.

[7] Yirmiya R, Goshen I (2011). Immune modulation of learning, memory, neural plasticity and neurogenesis. Brain Behav Immun.

[8] Lazosky A, Young GB, Zirul S, Phillips R (2010). Quality of life after septic illness. J Crit Care.

[9] Cheng WH (2019). CHIMERA repetitive mild traumatic brain injury induces chronic behavioural and neuropathological phenotypes in wild-type and APP/PS1 mice. Alzheimers Res Ther.

[10] Kissela B (2009). Clinical prediction of functional outcome after ischemic stroke: the surprising importance of periventricular white matter disease and race. Stroke.

[11] Cui X (2015). Deficiency of brain ATP-binding cassette transporter A-1 exacerbates blood-brain barrier and white matter damage after stroke. Stroke.

[12] Rodriguez-Grande B (2018). Gliovascular changes precede white matter damage and long-term disorders in juvenile mild closed head injury. Glia.

[13] Qin C (2017). Fingolimod protects against ischemic white matter damage by modulating microglia toward M2 polarization via STAT3 pathway. Stroke.

[14] Kaur C, Ling EA (2009). Periventricular white matter damage in the hypoxic neonatal brain: role of microglial cells. Prog Neurobiol.

[15] van Tilborg E (2016). Impaired oligodendrocyte maturation in preterm infants: potential therapeutic targets. Prog Neurobiol.

[16] Zhang Q (2019). The interleukin-4/PPARgamma signaling axis promotes oligodendrocyte differentiation and remyelination after brain injury. PLoS Biol.

[17] Ohtomo R, Iwata A, Arai K (2018). Molecular mechanisms of oligodendrocyte regeneration in white matter-related diseases. Int J Mol Sci.

[18] Kennaway DJ (2019). A critical review of melatonin assays: past and present. J Pineal Res.

[19] Lok R, van Koningsveld MJ, Gordijn MCM, Beersma DGM, Hut RA (2019). Daytime melatonin and light independently affect human alertness and body temperature. J Pineal Res.

[20] Phiphatwatcharaded C, Topark-Ngarm A, Puthongking P, Mahakunakorn P (2014). Anti-inflammatory activities of melatonin derivatives in lipopolysaccharide-stimulated RAW 264.7 cells and antinociceptive effects in mice. Drug Dev Res.

[21] Li Y (2019). Melatonin enhances autophagy and reduces apoptosis to promote locomotor recovery in spinal cord injury via the PI3K/AKT/mTOR signaling pathway. Neurochem Res.

[22] Tian X (2017). Melatonin promotes the in vitro development of microinjected pronuclear mouse embryos via its anti-oxidative and anti-apoptotic effects. Int J Mol Sci.

[23] Lai SW, Liu YS, Lu DY, Tsai CF (2019). Melatonin modulates the microenvironment of glioblastoma multiforme by targeting sirtuin 1. Nutrients.

[24] Hu Y (2017). Melatonin protects against blood-brain barrier damage by inhibiting the TLR4/NF-kappaB signaling pathway after LPS treatment in neonatal rats. Oncotarget.

[25] Rasheed MZ (2018). Melatonin improves behavioral and biochemical outcomes in a rotenone-induced rat model of Parkinson’s disease. J Environ Pathol Toxicol Oncol.

[26] Song J (2019). Pineal gland dysfunction in Alzheimer’s disease: relationship with the immune-pineal axis, sleep disturbance, and neurogenesis. Mol Neurodegener.

[27] Herzog-Krzywoszanska R, Krzywoszanski L (2019). Sleep disorders in Huntington’s disease. Front Psychiatry.

[28] Cecon E, Liu L, Jockers R (2019). Melatonin receptor structures shed new light on melatonin research. J Pineal Res.

[29] Jockers R (2016). Update on melatonin receptors: IUPHAR review 20. Br J Pharmacol.

[30] Han Q (2017). Microglia-derived IL-1beta contributes to axon development disorders and synaptic deficit through p38-MAPK signal pathway in septic neonatal rats. J Neuroinflammation.

[31] Smith EM, Pendlebury DF, Nandakumar J (2020). Structural biology of telomeres and telomerase. Cell Mol Life Sci.

[32] Zaret KS (2018). The telomerase enzyme and liver renewal. Nature.

[33] Liu MY, Nemes A, Zhou QG (2018). The emerging roles for telomerase in the central nervous system. Front Mol Neurosci.

[34] Spilsbury A, Miwa S, Attems J, Saretzki G (2015). The role of telomerase protein TERT in Alzheimer’s disease and in tau-related pathology in vitro. J Neurosci.

[35] Klapper W, Shin T, Mattson MP (2001). Differential regulation of telomerase activity and TERT expression during brain development in mice. J Neurosci Res.

[36] Lee J (2010). Telomerase deficiency affects normal brain functions in mice. Neurochem Res.

[37] Iannilli F, Zalfa F, Gartner A, Bagni C, Dotti CG (2013). Cytoplasmic TERT associates to RNA granules in fully mature neurons: role in the translational control of the cell cycle inhibitor p15INK4B. PLoS ONE.

[38] Raj DD (2015). Enhanced microglial pro-inflammatory response to lipopolysaccharide correlates with brain infiltration and blood-brain barrier dysregulation in a mouse model of telomere shortening. Aging Cell.

[39] Kronenberg G (2017). Repression of telomere-associated genes by microglia activation in neuropsychiatric disease. Eur Arch Psychiatry Clin Neurosci.

[40] Kang HJ (2004). Ectopic expression of the catalytic subunit of telomerase protects against brain injury resulting from ischemia and NMDA-induced neurotoxicity. J Neurosci.

[41] Zhang B (2010). Deficiency of telomerase activity aggravates the blood-brain barrier disruption and neuroinflammatory responses in a model of experimental stroke. J Neurosci Res.

[42] Khan AM (2015). Telomere dysfunction reduces microglial numbers without fully inducing an aging phenotype. Neurobiol Aging.

[43] Lu JJ (2016). Melatonin inhibits AP-2beta/hTERT, NF-kappaB/COX-2 and Akt/ERK and activates caspase/Cyto C signaling to enhance the antitumor activity of berberine in lung cancer cells. Oncotarget.

[44] Leon-Blanco MM, Guerrero JM, Reiter RJ, Calvo JR, Pozo D (2003). Melatonin inhibits telomerase activity in the MCF-7 tumor cell line both in vivo and in vitro. J Pineal Res.

[45] Dahlstrom J (2020). JAK2 inhibition in JAK2(V617F)-bearing leukemia cells enriches CD34(+) leukemic stem cells that are abolished by the telomerase inhibitor GRN163L. Biochem Biophys Res Commun.

[46] Huang P (2020). Complement C3a induces axonal hypomyelination in the periventricular white matter through activation of WNT/beta-catenin signal pathway in septic neonatal rats experimentally induced by lipopolysaccharide. Brain Pathol.

[47] Rezaie P, Dean A (2002). Periventricular leukomalacia, inflammation and white matter lesions within the developing nervous system. Neuropathology.

[48] McQuillen PS, Ferriero DM (2004). Selective vulnerability in the developing central nervous system. Pediatr Neurol.

[49] Ness JK, Romanko MJ, Rothstein RP, Wood TL, Levison SW (2001). Perinatal hypoxia-ischemia induces apoptotic and excitotoxic death of periventricular white matter oligodendrocyte progenitors. Dev Neurosci.

[50] Lin L (2019). Synaptic structure and alterations in the hippocampus in neonatal rats exposed to lipopolysaccharide. Neurosci Lett.

[51] Lin Q (2019). Interleukin-1beta disturbs the proliferation and differentiation of neural precursor cells in the hippocampus via activation of notch signaling in postnatal rats exposed to lipopolysaccharide. ACS Chem Neurosci.

[52] Chung YC (2017). Capsaicin prevents degeneration of dopamine neurons by inhibiting glial activation and oxidative stress in the MPTP model of Parkinson’s disease. Exp Mol Med.

[53] Carloni S, Riparini G, Buonocore G, Balduini W (2017). Rapid modulation of the silent information regulator 1 by melatonin after hypoxia-ischemia in the neonatal rat brain. J Pineal Res.

[54] Gong YH, Hua N, Zang X, Huang T, He L (2018). Melatonin ameliorates Abeta1-42 -induced Alzheimer’s cognitive deficits in mouse model. J Pharm Pharmacol.

[55] Tsai TH (2017). Melatonin attenuated the brain damage and cognitive impairment partially through MT2 melatonin receptor in mice with chronic cerebral hypoperfusion. Oncotarget.

[56] Chen LY (2017). Melatonin successfully rescues hippocampal bioenergetics and improves cognitive function following drug intoxication by promoting Nrf2-ARE signaling activity. J Pineal Res.

[57] Olivier P (2009). Melatonin promotes oligodendroglial maturation of injured white matter in neonatal rats. PLoS ONE.

[58] Kaur C, Sivakumar V, Ling EA (2010). Melatonin protects periventricular white matter from damage due to hypoxia. J Pineal Res.

[59] Yamada O, Kawauchi K (2013). The role of the JAK-STAT pathway and related signal cascades in telomerase activation during the development of hematologic malignancies. JAKSTAT.

