# Supplementary figures and images for: Melatonin Reduces Neuroinflammation and Improves Axonal Hypomyelination by Modulating M1/M2 Microglia Polarization via JAK2-STAT3-Telomerase Pathway in Postnatal Rats Exposed to Lipopolysaccharide
Source: Mol Neurobiol. 2021 Sep 28;58(12):6552–76. doi: 10.1007/s12035-021-02568-7 (PMC8639545; doi:10.1007/s12035-021-02568-7)

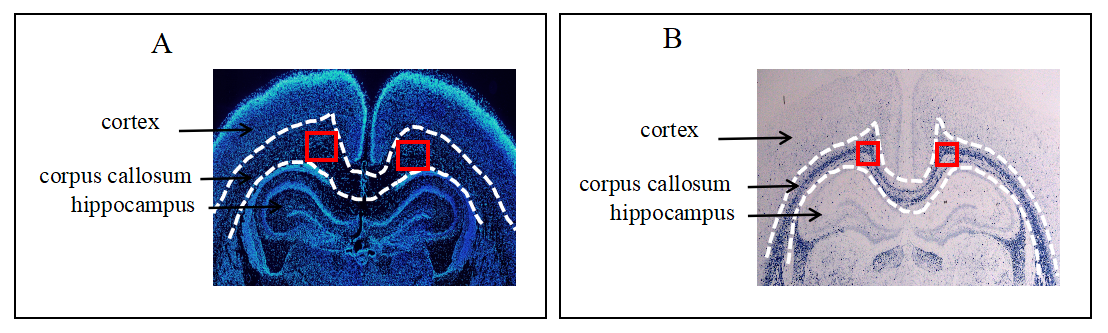

Supplement: Supplementary file 1 — Supporting Fig. 1 The overall status image of corpus callosum. A (immunofluorescence) and B (in situ hybridization) showed the area of the corpus callosum analyzed in this study. The white box shows the corpus callosum in brain. The red box shows the lesion area of the corpus callosum as analyzed in this study. Supplementary file1 (TIF 1460 kb) [file 12035_2021_2568_MOESM1_ESM.tif]

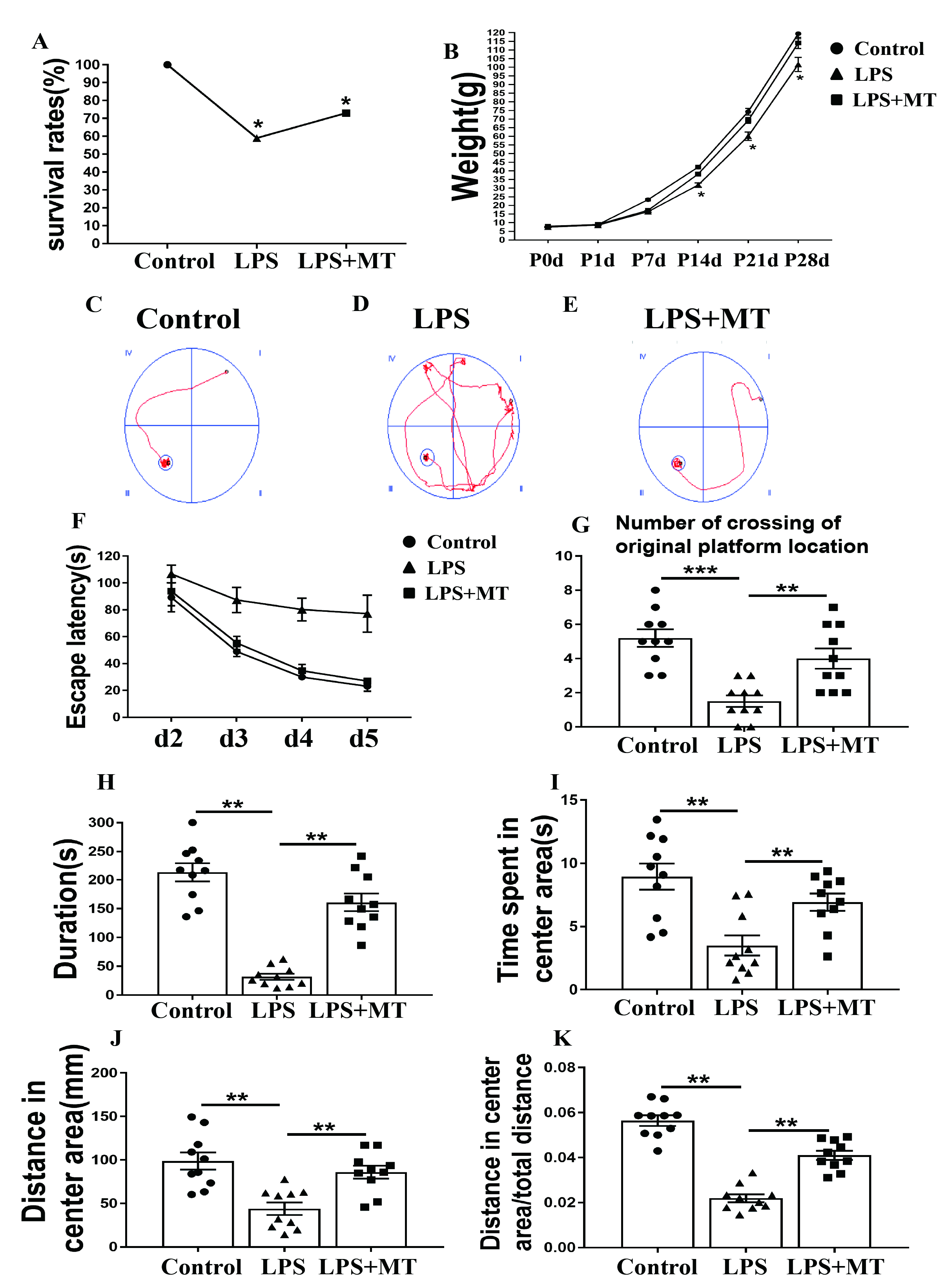

Supplement: Supplementary file 2 — Supporting Fig. 2 Melatonin improved behavioral performance in the LPS injected rat model. The survival rate (A) and body weight of rats (B) following LPS injection with or without melatonin treatment and their corresponding controls. (C-G) Cognitive performance in the Morris water maze task. The rats at 28d in LPS + melatonin group showed shorter escape latencies (F) and increased number of times crossing the original platform location (G) than the LPS group. (H)The rotarod test showed that melatonin treatment significantly improved the impairments in motor deficits of animals after LPS injection as evident by the longer duration staying on the rotarod. (I-K) The effects of melatonin on behaviors in the open field test. In LPS + melatonin group, the recorded time (I) and distance (J) in the center area were longer than the LPS group. In LPS + melatonin group the ratio between distance in the center arena and the total distance (K) was increased than the LPS group. *P<0.05, **P<0.01, n=10 for each group in every test. Supplementary file2 (TIF 31211 kb) [file 12035_2021_2568_MOESM2_ESM.tif]

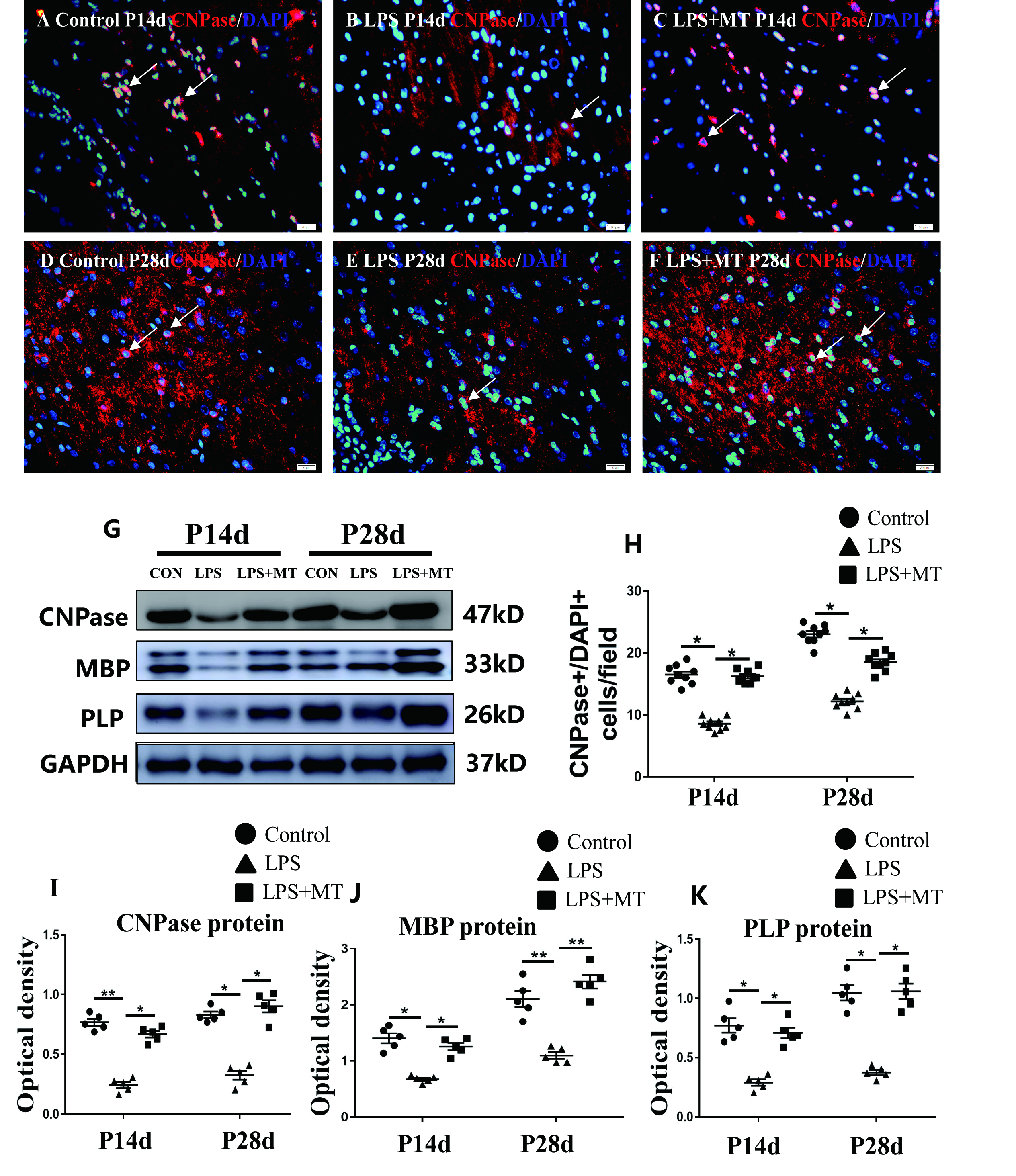

Supplement: Supplementary file 3 — Supporting Fig. 3 Melatonin reversed the decline in expression of myelin associated proteins in the corpus callosum of postnatal rats at 14 and 28d after LPS injection. (A-F) CNPase immunofluorescence in the corpus callosum in postnatal rats at 14 and 28d after LPS (B, E) or LPS + melatonin (C, F) injection and their corresponding controls (A, D). Bar H summarized the frequency of CNPase+/DAPI+ cells at 14 and 28d after LPS/melatonin injection when compared with their corresponding controls (n=5 for each group). (G)Western blot analysis of PLP, MBP and CNPase protein expression levels in the corpus callosum of postnatal rats at 14 and 28d after LPS or LPS + melatonin injection and their matching controls. Graph I-K depicted the optical density changes of CNPase, PLP and MBP, respectively, relative to GAPDH, (n=5 for each group). It is evident that melatonin can reverse the decreased expression of CNPase, PLP and MBP protein in the corpus callosum induced by LPS exposure at 14 and 28d. Scale bars: A-F 20µm. *P<0.05, **P < 0.01. Supplementary file3 (TIF 37790 kb) [file 12035_2021_2568_MOESM3_ESM.tif]

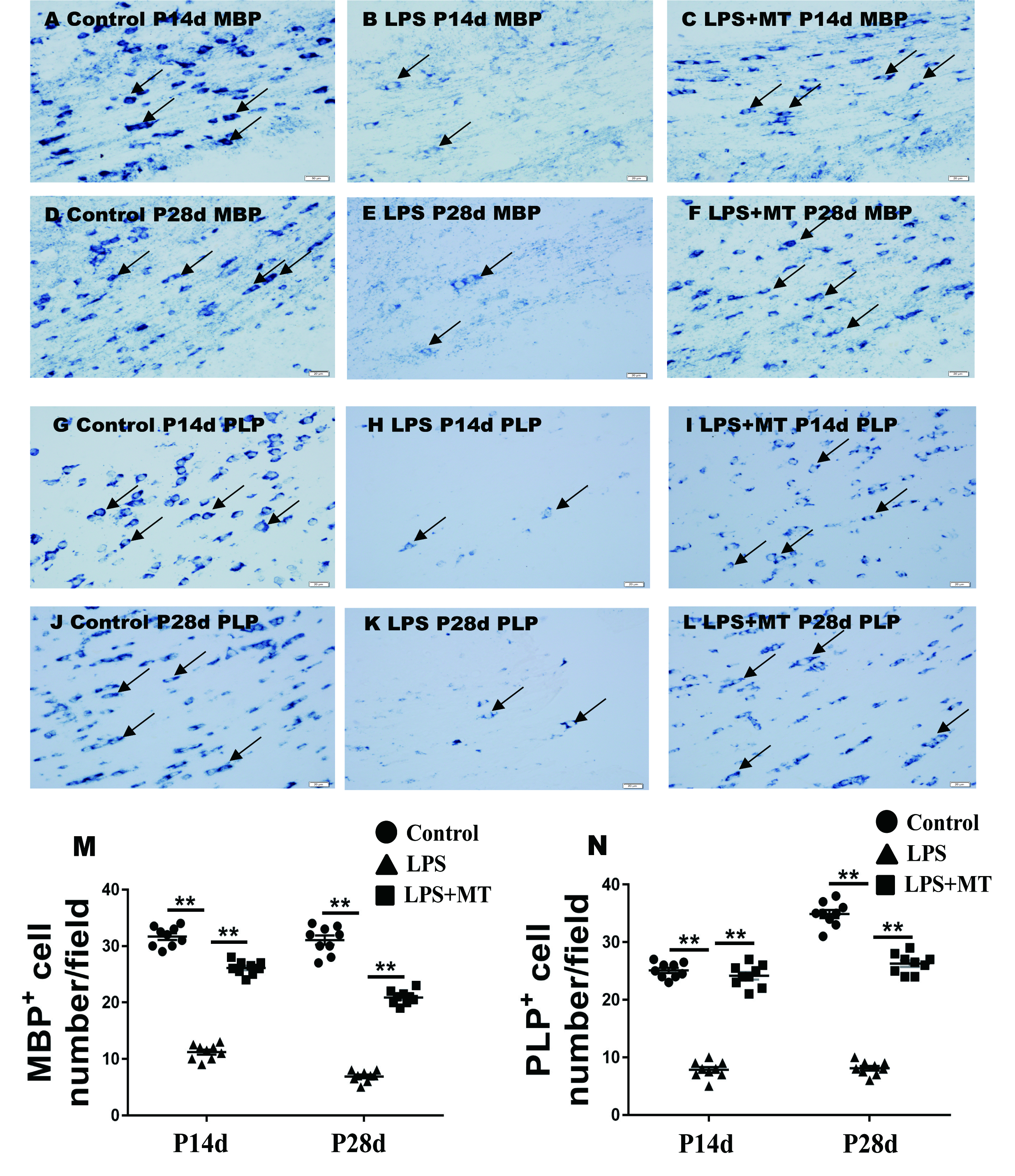

Supplement: Supplementary file 4 — Supporting Fig. 4 Melatonin increased the cell numbers of PLP+ and MBP+ oligodendrocytes in the corpus callosum after LPS injection as revealed by in situ hybridization. (A-L) In situ hybridization shows the number of PLP+ and MBP+ oligodendrocytes in the corpus callosum at 14 and 28d after LPS injection (B, E, H, K), LPS + melatonin administration (C, F, I, L) and the corresponding controls (A, D, G, J) at the magnification of ×40. Note melatonin treatment reverses the decreased number of PLP+ and MBP+ oligodendrocytes in the corpus callosum induced by LPS exposure at 14 and 28d. Bar graph (M, N) summarized the frequency of PLP+ and MBP+ oligodendrocytes in the corpus callosum at 14 and 28d using in situ hybridization. Scale bars: A-L 20µm. *P < 0.05, **P < 0.01, n=5 for each group. Supplementary file4 (TIF 40700 kb) [file 12035_2021_2568_MOESM4_ESM.tif]

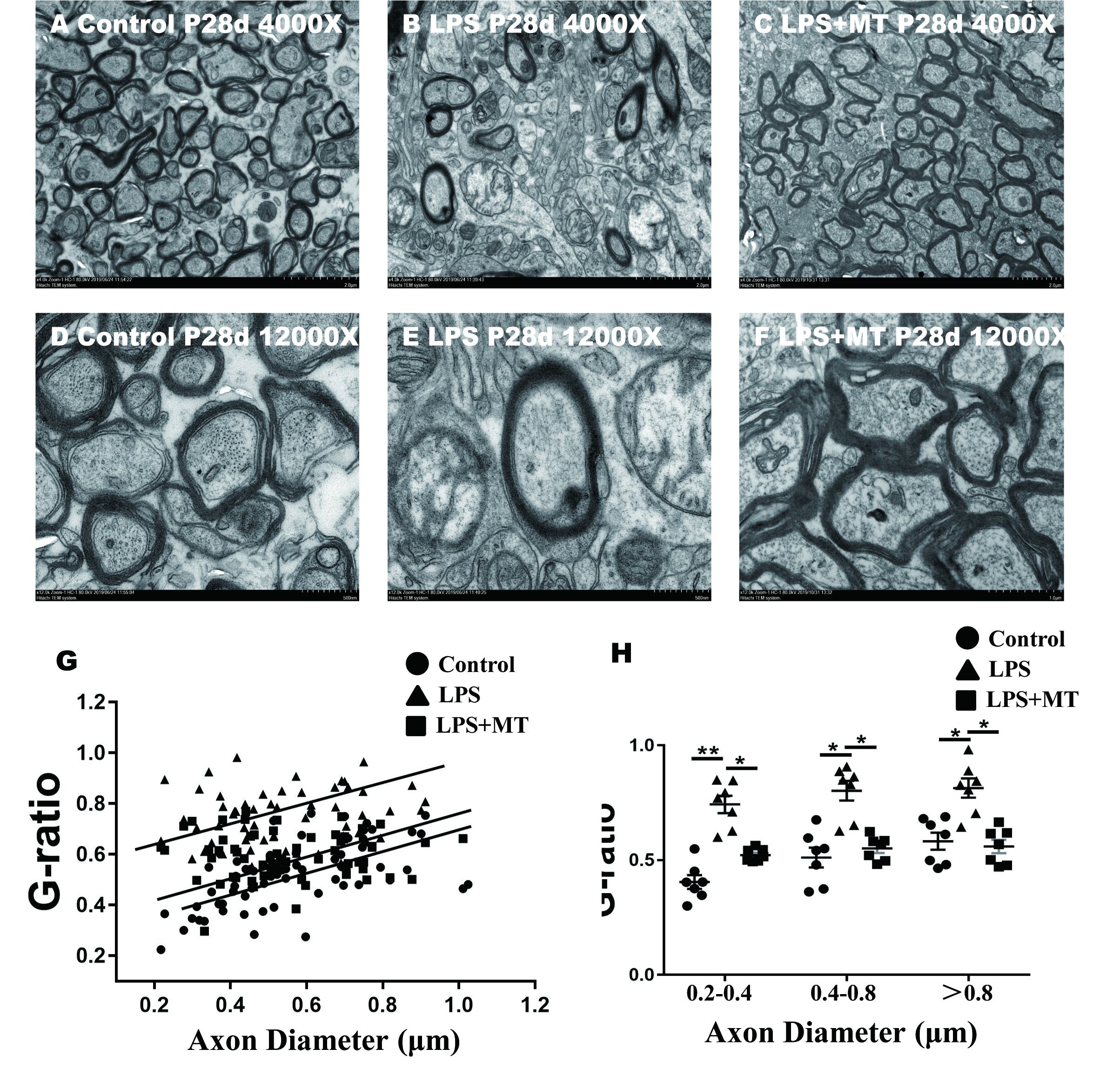

Supplement: Supplementary file 5 — Supporting Fig. 5 Melatonin prevented hypomyelination in the corpus callosum after LPS injection as revealed by electron microscopy. Electron microscopic images of corpus callosum at the magnification of ×4000 (A-C) and ×12000 (D-F). A-F show transverse section of myelinated axons in the corpus callosum at 28d after LPS (B, E) and LPS + melatonin injection (C, F) and their corresponding controls (A, D). Scatter diagram of g-ratio to axon diameter in the corpus callosum at 28d after LPS and LPS + melatonin injection and their matching controls were shown in bar graph G. H is bar graph showing g-ratio of myelinated axons of different diameters in the corpus callosum at 28d after LPS and LPS + melatonin injection and corresponding control. Scale bars: A-C 2 µm, D-F 500nm. *P < 0.05, **P < 0.01, n=5 for each group. Supplementary file5 (TIF 32291 kb) [file 12035_2021_2568_MOESM5_ESM.tif]

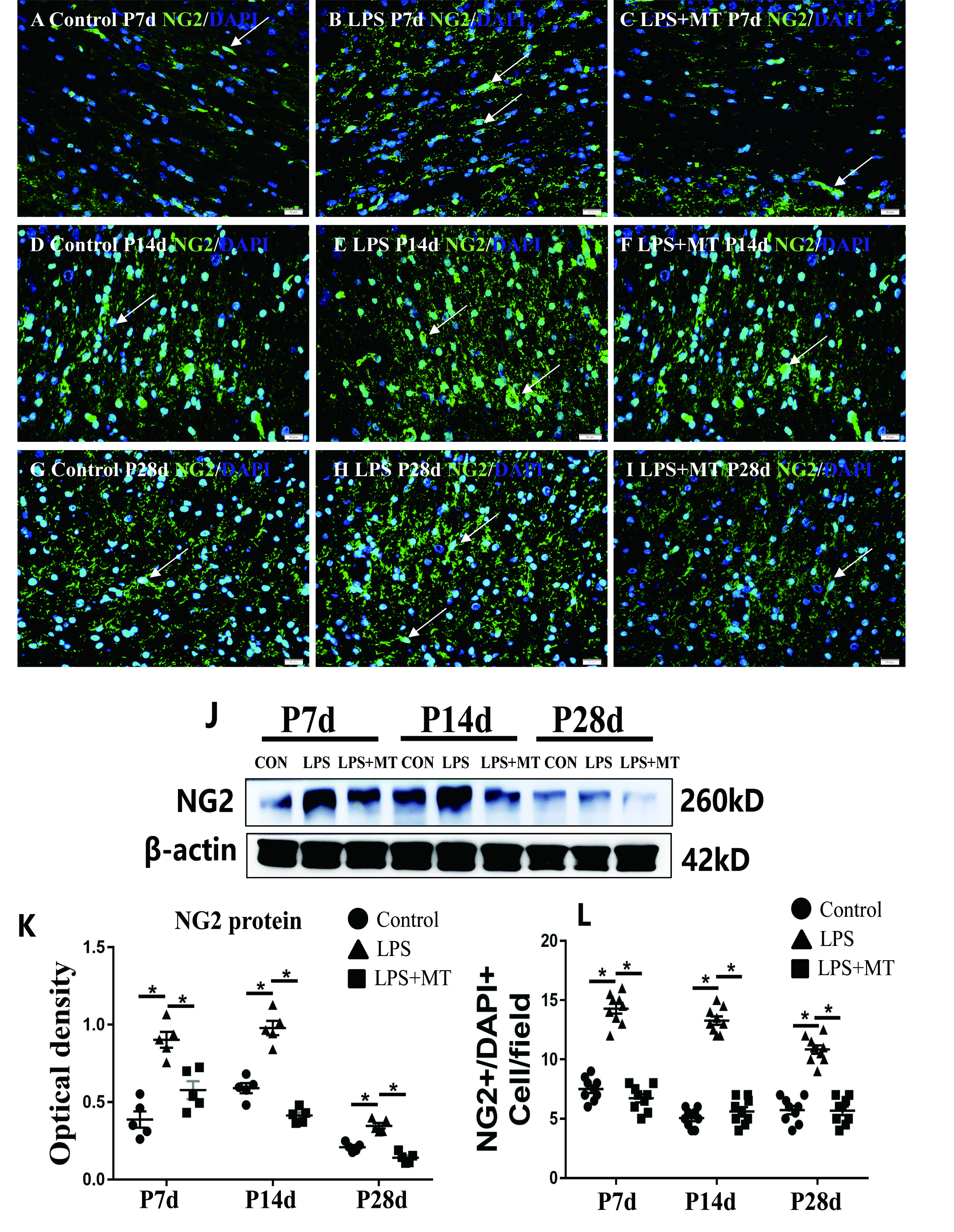

Supplement: Supplementary file 6 — Supporting Fig. 6 Effect of melatonin on the differentiation and maturation of OPCs in the corpus callosum of postnatal rats after LPS/melatonin injection. Immunofluorescence staining showing NG2 labelled oligodendrocytes (green) and DAPI (blue) in the corpus callosum in postnatal rats at 7, 14 and 28d after LPS (B, E, H) and LPS + melatonin injection (C, F, I) and their corresponding controls (A, D, G) at the magnification of ×40. (L) Bar graphs show the number of NG2+/DAPI+ in the corpus callosum at 7, 14 and 28d after LPS injection, LPS + melatonin administration and their matching controls (n=5 for each group). (J) Western blot analysis of NG2 expression levels in the corpus callosum in postnatal rats at 7, 14 and 28d after LPS/melatonin injection and their matching controls. Graph K showed optical density changes of NG2 relative to β-actin (n=5 for each group). Scale bars: 20 µm. *P < 0.05, **P < 0.01. Supplementary file6 (TIF 42352 kb) [file 12035_2021_2568_MOESM6_ESM.tif]

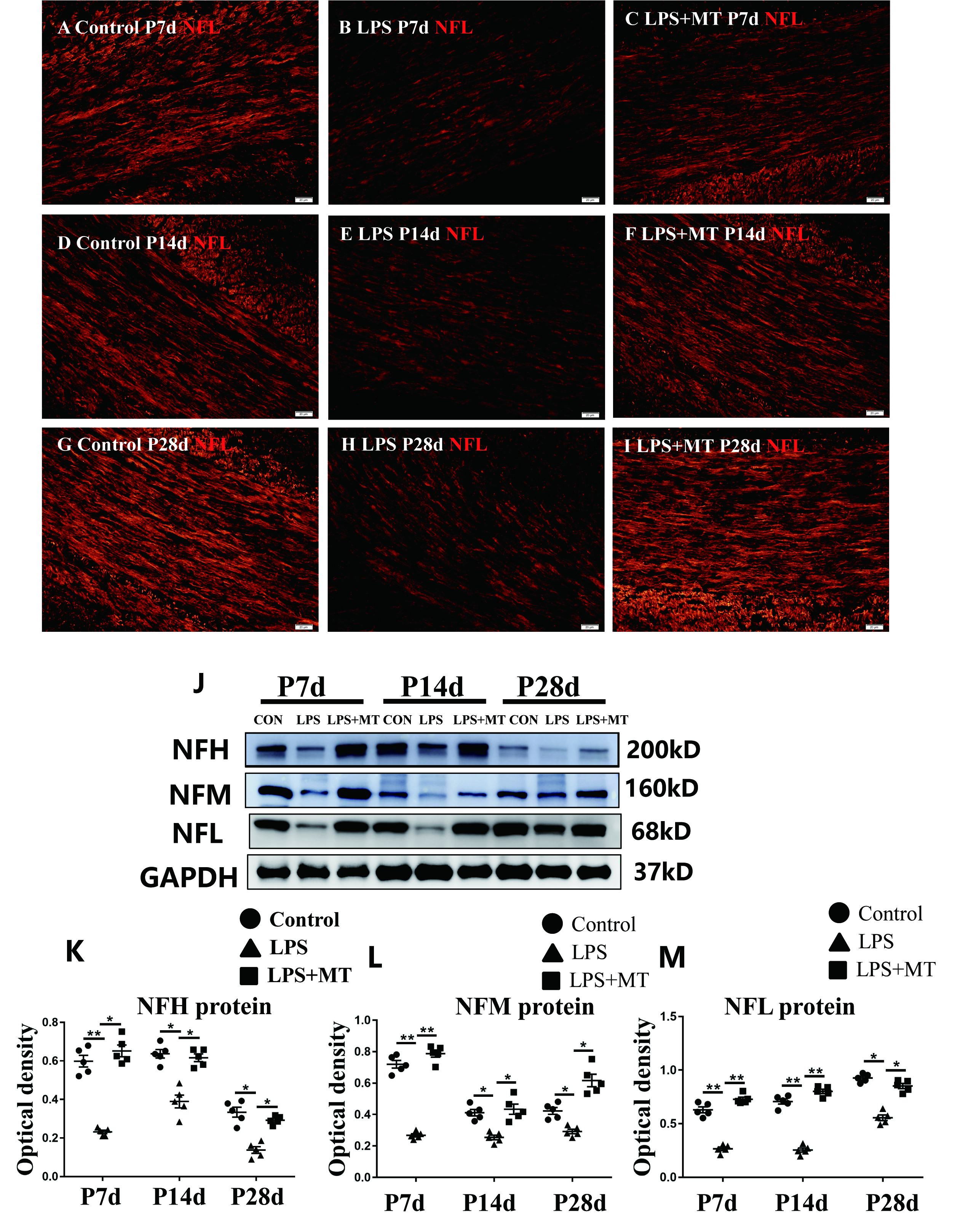

Supplement: Supplementary file 7 — Supporting Fig. 7 Melatonin upregulated the expression of neurofilament proteins NFL, NFM and NFH expression which played an important role in the axonal caliber of myelinated axons. Immunofluorescence staining shows NFL immunofluorescence (red) and DAPI (blue) in the corpus callosum in postnatal rats at 7, 14 and 28d after LPS (B, E, H) and LPS + melatonin injection (C, F, I) and their matching controls (A, D, G) at the magnification of ×40 (n=5 for each group). Panel J shows NFL, NFM, and NFH immunoreactive bands. (K-M) Bar graphs depict the optical density of NFL, NFM, and NFH expression shown in J. Quantification by immunoblot (J) shows significant decrease in NFL, NFM, and NFH protein expression at 7, 14 and 28d after LPS treatment in comparison with control; however, melatonin administration upregulates the expression of NFL, NFM, and NFH protein expression significantly (n=5 for each group). Scale bars: 20 μm. *P < 0.05, **P < 0.01. Supplementary file7 (TIF 44814 kb) [file 12035_2021_2568_MOESM7_ESM.tif]

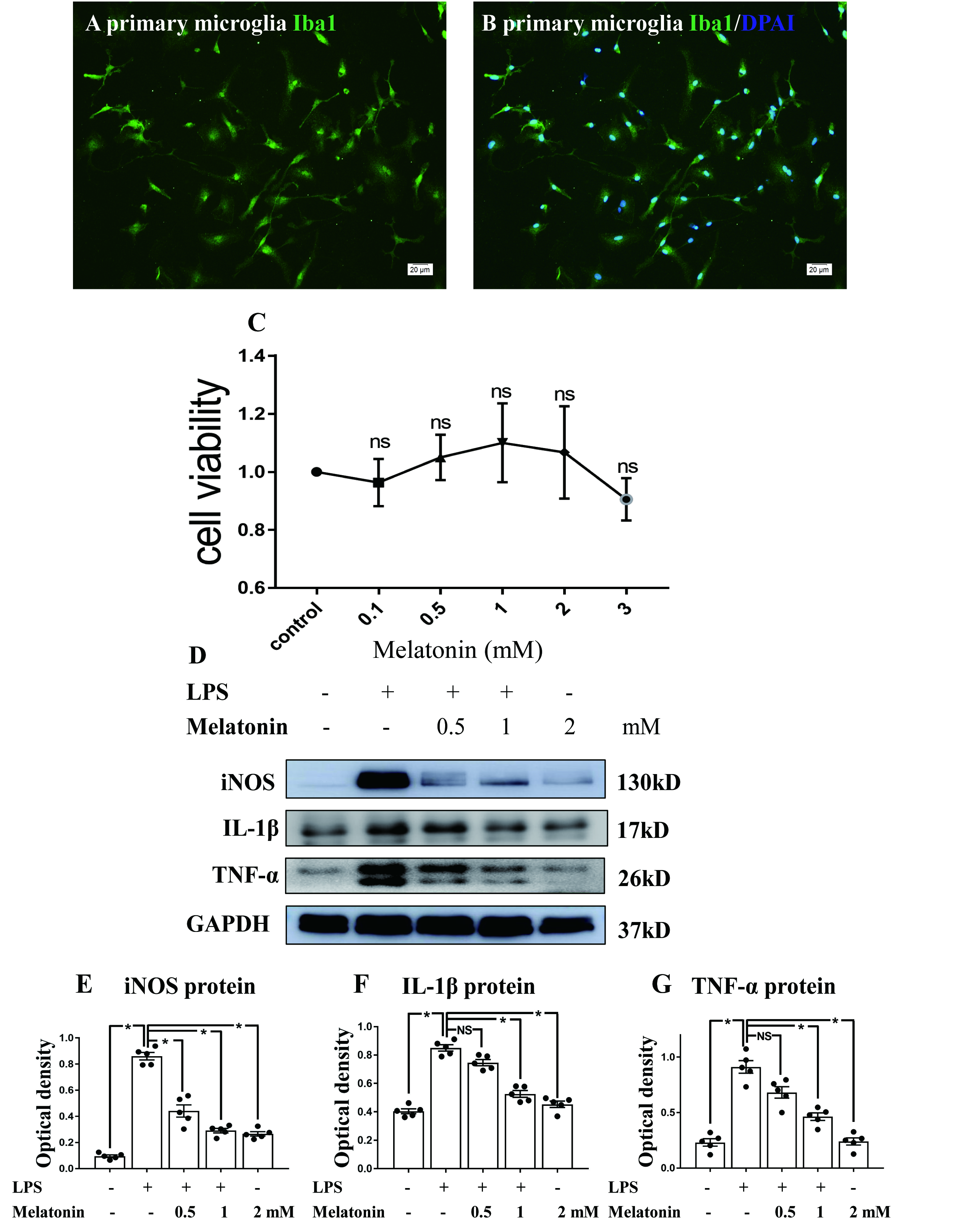

Supplement: Supplementary file 8 — Supporting Fig. 8 Microglia purity test and the effect of different concentrations of melatonin on microglia viability and the optimal working concentration of melatonin. Iba1 was used as a marker for microglia, and DAPI was used for nucleus staining. Microglia cultures over 98% purity were used in this study. (A, B) CCK-8 assay showed microglia viability treated with a concentration gradient of melatonin at 0, 0.1, 0.5, 1, 2, 3 mM for 24h (graph A). Panel B shows pro-inflammatory mediators, including iNOS (130kDa), TNF-α (26kDa), IL-1β (17kDa) and GAPDH (37kDa) immunoreactive bands after the LPS and LPS + different concentrations of melatonin (0.5 mM, 1 mM and 2 mM) treatment when compared with the corresponding control in primary microglia. Bar graphs in B-E show optical density changes of iNOS, TNF-α and IL-1β relative to GAPDH of each group. For this study, 1mM melatonin was adopted as the optimal working concentration to treat microglia in different experiments. Scale bars: * P<0.05, n=5 for each group. Supplementary file8 (TIF 40668 kb) [file 12035_2021_2568_MOESM8_ESM.tif]

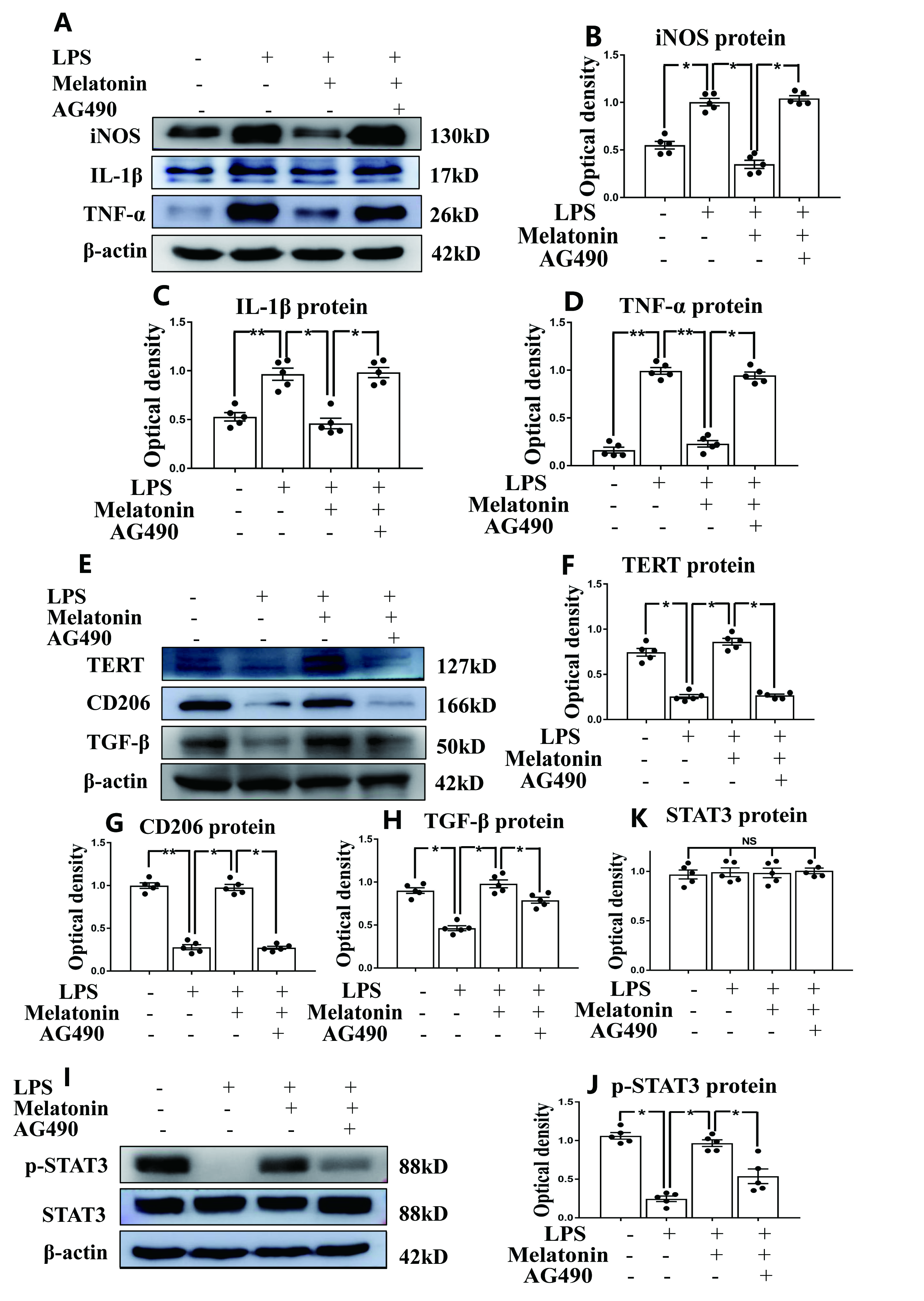

Supplement: Supplementary file 9 — Supporting Fig. 9. Melatonin modulates microglial polarization from M1 to M2 phenotype through JAK2/STAT3 pathway in vitro. Panel A shows pro-inflammatory mediators, including iNOS (130kDa), TNF-α (26kDa), IL-1β (17kDa) and GAPDH (37kDa) immunoreactive bands. Panel E shows anti-inflammatory mediators, including CD206 (166kDa), TGF-β (50kDa), TERT (127kDa) and GAPDH (37kDa). Panel I shows p-STAT3 (88kDa), STAT3 (88kDa) and GAPDH (37kDa) immunoreactive bands after LPS, melatonin or AG490 (an inhibitor of JAK2 activity) treatment when compared with the corresponding control in primary microglia. Bar graphs in B-D, F-H and J-K show optical density changes of iNOS, TNF-α, IL-1β, CD206, TGF-β, p-STAT3, STAT3 relative to GAPDH of each group. Note melatonin modulates the conversion of M1 to M2 polarization through JAK2/STAT3 pathway. Scale bars: * P<0.05, n=5 for each group. Supplementary file9 (TIF 38962 kb) [file 12035_2021_2568_MOESM9_ESM.tif]

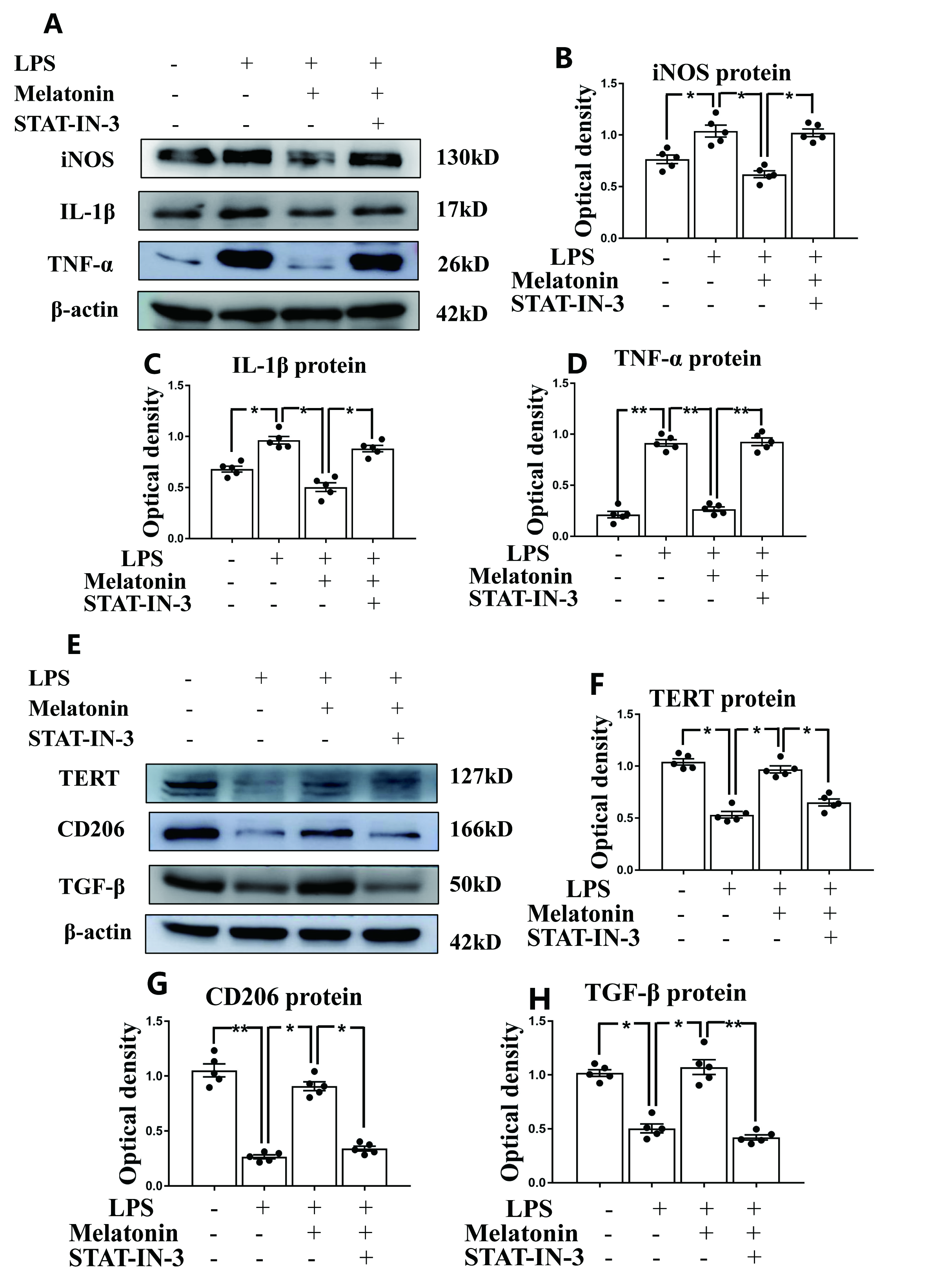

Supplement: Supplementary file 10 — Supporting Fig. 10. Melatonin modulates microglial polarization from M1 to M2 phenotype through JAK2/STAT3 pathway in vitro. Panel A shows pro-inflammatory mediators, including iNOS (130kDa), TNF-α (26kDa), IL-1β (17kDa) and GAPDH (37kDa) immunoreactive bands and panel E shows anti-inflammatory mediators, including CD206 (166kDa), TGF-β (50kDa), TERT (127kDa) and GAPDH (37kDa) immunoreactive bands after the LPS, melatonin or STAT-IN-3 (an inhibitor of STAT3 activity) treatment when compared with the corresponding control in primary microglia. Bar graphs in B-D and F-H show optical density changes of iNOS, TNF-α, IL-1β, CD206 and TGF-β relative to GAPDH of each group. Note melatonin treatment modulates the conversion of M1 to M2 polarization through JAK2/STAT3 pathway. Scale bars: * P<0.05, n=5 for each group.Supplementary file10 (TIF 35281 kb) [file 12035_2021_2568_MOESM10_ESM.tif]
